# Targeting PLCG2 Suppresses Tumor Progression, Orchestrates the Tumor Immune Microenvironment and Potentiates Immune Checkpoint Blockade Therapy for Colorectal Cancer

**DOI:** 10.7150/ijbs.98200

**Published:** 2024-10-14

**Authors:** Xueliang Zhou, Joshua Lin, Yanfei Shao, Huang Zheng, Yi Yang, Shuchun Li, Xiaodong Fan, Hiju Hong, Zhihai Mao, Pei Xue, Sen Zhang, Jing Sun

**Affiliations:** 1 Department of General Surgery, Ruijin Hospital, Shanghai Jiao Tong University School of Medicine, Shanghai, China.; 2 Shanghai Minimally Invasive Surgery Center, Ruijin Hospital, Shanghai Jiao Tong University School of Medicine, Shanghai, China.; 3 Shanghai Institute of Digestive Surgery, Ruijin Hospital, Shanghai Jiao Tong University School of Medicine, Shanghai, China.; 4 Department of General Surgery, Wuxi branch of Ruijin Hospital Shanghai Jiao Tong University School of Medicine, Wuxi, China.

**Keywords:** Phospholipase Cγ2 (PLCG2), Colorectal cancer, Tumor progression, Tumor immune microenvironment, Immune checkpoint blockade therapy, Therapeutic target

## Abstract

**Background:** Tumor progression and limited benefits of immune checkpoint blockade (ICB) therapy have been two major challenges in the clinical management of colorectal cancer (CRC). The objective of our research was to explore the role of PLCG2 in CRC progression, tumor microenvironment, and potentiating ICB therapy.

**Methods:** Based on bioinformatics analysis and a prospective clinical observational study, the expression, prognostic significance, and clinical relevance of PLCG2 in CRC were unveiled. The single-cell and spatial transcriptome revealed the role of PLCG2 in shaping the heterogeneity of the CRC tumor microenvironment. The biological function of PLCG2 was validated by *in vivo* and *in vitro* experiments. The underlying mechanisms were elucidated by RNA-seq, western blotting, qRT-PCR, and multicolor immunofluorescence. The multiplex immunohistochemistry and flow cytometry were adopted to clarify the immunomodulatory role of PLCG2 in facilitating CRC immune escape. The translational value of targeting PLCG2 to potentiate the efficacy of ICB therapy and synergistic therapy to improve prognosis was explored in the preclinical animal models.

**Results:** In CRC, PLCG2 exhibited high expression levels and was strongly associated with poor prognosis and advanced clinicopathological characteristics of patients. The single-cell transcriptome shed light on its important role in cell communication and the development and differentiation of immune cells. The spatial transcriptome described the spatial distribution of PLCG2 in CRC tissues. Further mechanistic analysis demonstrated that PLCG2 could promote proliferation, invasion, metastasis, epithelial-mesenchymal transition, and cell cycle regulation and inhibit apoptosis of CRC cells via the Akt-mTOR pathway activation. Furthermore, PLCG2 was found to contribute greatly to the immunosuppressive microenvironment and enhanced immune escape as it significantly suppressed the infiltration and functional activation of CD8^+^ T cells and promoted the infiltration of Treg cells as well as PD-1 and PD-L1 expression. Meanwhile, knockdown of PLCG2 could potentiate the efficacy of ICB therapy.

**Conclusion:** In summary, we have identified for the first time that PLCG2 could be considered a precise biomarker and promising therapeutic target for predicting CRC prognosis, optimizing individualized treatment, reversing CRC immune escape, and overcoming resistance to ICB therapy.

## Introduction

According to statistical analysis, there were 1,926,118 new cases of CRC globally in 2022, which ranked third in overall incidence, third among male malignant tumor cases, and third among female malignant tumor cases. There were about 0.90 million deaths of CRC globally in 2022, which ranked second in overall mortality, and global deaths continued to rise annually 1. The current treatment for CRC is a multidisciplinary approach revolving around surgical resection, comprising targeted therapy, neoadjuvant and postoperative radiotherapy, and immunotherapy. This comprehensive strategy has notably enhanced the prognosis and outcomes for CRC patients. Nonetheless, for advanced CRC patients, especially those who have developed distant metastases, the survival rate is still low, showing a five-year survival rate of less than 10% in some patients 2, 3. Therefore, the research of novel and reliable biomarkers and therapeutic targets for CRC, as well as the investigation of the underlying biological molecular mechanisms, are urgent and crucial to improving patient prognosis.

Phospholipase C (PLC) is an imperative enzyme in the phosphatidylinositol signal transduction pathway 4. Endogenous PLCs are considered important substances for intracellular signaling and interact with other intracellular signaling pathways to form an incredibly complex signaling regulatory network. Currently, it is well established that mammals possess 12 PLC isozymes, which can be categorized into 6 major groups by their distinct structures and regulatory mechanisms. The PLCG2 gene is responsible for encoding phospholipase Cγ2. PLCγ2 is activated in three main forms: tyrosine phosphorylation, direct protein interactions with Rac GTPase, and conformational changes mediated by the SH2 region 5. Activated PLCγ2 regulates the development of various cells and their physiological functions and drives numerous pathological and physiological events such as tumors, immunity, inflammation, and allergic reactions. Recent studies have reported the integral role PLCG2 plays in developing malignant tumors. PLCγ2 signaling has been reported to drive leukemia in some cases. Ibrutinib emerged as an important and effective treatment for chronic lymphocytic leukemia (CLL) by inhibiting BTK and thereby blocking the PLCγ2 signaling pathway 6. In non-small cell lung cancer, PLCG2, an important oncogene and prognostic biomarker, was involved in tumor cell metastasis through the regulation of mitochondrial respiration 7. Li *et al.* found that PLCG2 could be an effective biomarker for predicting the prognosis and immune status of the tumor microenvironment in patients with soft-tissue sarcoma (STS), which provided important insights into understanding the role of PLCG2 in immune remodeling of the tumor microenvironment 8. It was noteworthy that the role of PLCG2 in CRC has rarely been investigated, and its relationship with the tumor microenvironment (TME) remained poorly understood.

Rapid advances in bioinformatics analysis methods have provided unprecedented opportunities to discover novel and reliable biomarkers and therapeutic targets 9. In order to deeply resolve the biological roles and molecular mechanisms of PLCG2 in CRC, the present study employed a variety of bioinformatics analyses aimed at comprehensively revealing the biological function and mechanism of action of PLCG2 in tumor progression and tumor microenvironment. The implication of PLCG2 expression on the survival of CRC patients could be assessed by Kaplan-Meier (KM) survival analysis, providing important information for clinical prognostic assessment 10. Then, genomic mutation analysis revealed the mutational spectrum of the PLCG2 gene, which helped understand its role in tumorigenesis at the molecular level 11. Single-cell sequencing analysis enabled us to identify PLCG2 expression patterns in different tumor cell subpopulations and their interactions with other genes with a high-resolution view of the cellular heterogeneity in the tumor microenvironment. Spatial transcriptome sequencing analysis further complemented the single-cell sequencing technology by offering valuable insights into the spatial distribution of cells in tumor tissues, contributing to understanding the spatial heterogeneity of PLCG2 in the tumor microenvironment 12. Multiple algorithms, such as CIBERSORT, were invaluable tools for exploring the PLCG2-regulated composition and state of immune cells in CRC 13,14. The integrated application of these bioinformatic methods not only deepened the understanding of the role of PLCG2 in CRC and the tumor microenvironment but also provided new strategies and molecular targets for CRC.

In the study, we conducted a comprehensive analysis to reveal the role of PLCG2 as an important pro-oncogene in promoting CRC progression, forming an immunosuppressive microenvironment, and predicting immunotherapy sensitivity. A prospective clinical observational study based on an independent CRC cohort from our medical center was carried out to confirm that PLCG2 was a risk factor for the outcome of CRC patients and was closely related to clinicopathological features (Clinical Trial Registry No. NCT04714814). The single-cell transcriptome shed light on its important role in cell communication and the development and differentiation of immune cells. The spatial transcriptome described the spatial distribution of PLCG2 in CRC tissues. Further molecular mechanisms and rescue experiments confirmed that high expression of PLCG2 promoted CRC cell proliferation, epithelial-mesenchymal transition (EMT), migration, and invasion and inhibited apoptosis by activating the Akt-mTOR signaling pathway. This study also elucidated that PLCG2 was closely tied to the development of immunosuppressive microenvironment. Additionally, knockdown of PLCG2 potentiated the efficacy of anti-PD-1 therapy for CRC, and targeted inhibition of PLCG2 in combination with immune checkpoint blockade (ICB) therapy might be an effective strategy to improve the immunotherapy response and survival of CRC patients. Therefore, PLCG2 was believed for the first time to be a novel biomarker and therapeutic target for CRC with great potential.

## Material and methods

### Preparation of CRC tissue microarray and patient follow-up

Tissue samples were collected from 76 patients with pathologically diagnosed CRC without any preoperative treatment for tissue microarrays preparation. The clinicopathological information of the patients was collected to construct the clinical database, and the cohort was followed up by the dedicated person, which was named the Ruijin cohort. The following inclusion criteria must all be met for eligibility: (1) Age ≥18 years and ≤70 years; (2) ECOG performance status of 0 or 1; (3) Pathologically proven colorectal adenocarcinoma; (4) No family history of hereditary cancer; (5) Treated with standardized radical resection of colorectal cancer satisfying R0 resection and removal of at least 12 lymph nodes; (6) Adequate primary tumor tissues from surgery or biopsy in compliance with the sample criteria; (7) Adequate peripheral blood sample in compliance with the sample criteria; (8) Patient able and voluntary to provide legitimate informed consent for the study. The exclusion criteria included the following : (1) Currently participating in clinical trials that might interfere with treatment judgments or receiving experimental treatments; (2) Patients who present abnormalities involving heart, lung, liver, kidney, hematopoiesis, and bone marrow reserve that cannot tolerate surgical treatments and chemotherapy; (3) Any other progressive malignant disease or malignancy requiring treatment within the preceding 5 years; (4) Mental illness or serious cardiovascular disease; (5) Pregnancy, breastfeeding, or planning for pregnancy within 1 year. Postoperative follow-up time points for CRC patients were based on specific time points after surgery, including scheduled follow-up visits at the first, third, and sixth months after surgery and subsequent six-month or annual follow-up visits. At each follow-up visit, high-quality clinical assessment, appropriate imaging and laboratory tests, and detailed data recording and analysis played important roles in evaluating treatment outcomes, monitoring survival, and guiding subsequent treatment decisions.

### Bioinformatics analysis

Transcriptomic data of pan-cancer and clinicopathological information were obtained from the official website of TCGA (https://portal.gdc.cancer.gov/). Subsequently, we conducted preprocessing steps, encompassing quality control assessments and normalization procedures for gene expression data. The clinical data of patients was integrated with gene expression data, and the Kaplan-Meier survival estimate was utilized to study the association of PLCG2 expression and prognosis. A diagnostic model was developed with the gene expression data in pan-cancer, and the diagnostic performance based on PLCG2 expression was assessed using the diagnostic curve ROC.

Six tumor tissue specimens (M1001, M1002, M1003, M1004, M1005 and M1006) pathologically diagnosed as adenocarcinoma undergoing radical colorectal surgery without any preoperative treatment at our center were collected, and the single cell transcriptome sequencing was performed, which was consistent with the inclusion and exclusion criteria for the prospective observational clinical study. After data integration, pre-processing, and rigorous quality control, the final 33,538 cells were collected for subsequent analyses. The detailed procedures of pre-processing and quality control were as follows: (1) Raw data quality control; (2) Data comparison and mapping; (3) Cell Barcode Correction; (4) UMI disaggregation; (5) Count matrix generation; (6) Quality control indicators; (7) Data standardization and normalization; (8) Identification of highly variable genes (HVGs); (9) Filtering condition settings: nCount_RNA > 1000 & nFeature_RNA < 3000 & percent.mt < 10 & nFeature_RNA > 200. The t-SNE algorithm was used for downscaling and cell clustering, and the "SingleR" R package was used for cell annotation. The "CellChat" and "NicheNet" R packages were used for cell communication. The "monocle2" was used for cell trajectory analysis.

Spatial transcriptome sequencing was also performed concurrently with patient M1002. Loupe Browser software was used for the analysis of the 10X Visium spatial transcriptome. The CellMarker database (http://biocc.hrbmu.edu.cn/CellMarker/) was used for cell annotation of spatial region subpopulations.

### Cell culture, cell transfection and drug treatment

RKO, HT-29, HCT116, SW480, SW620 and NCM460 cell lines were purchased from American Type Culture Collection (ATCC). Cells were cultured at 37°C in a constant temperature incubator with 5% carbon dioxide (CO_2_) to maintain optimal growth conditions. Observe and monitor the cell morphology and proliferation by microscope and pay close attention to the health of the cells. Cell identification and detection, including detection and identification of cell contamination (e.g., bacteria, fungi, mycoplasma, etc.) were performed regularly.

PLCG2 overexpression and knockdown lentiviruses were from Shanghai Genechem Co., Ltd. The fully packaged lentivirus was transfected with the target cell lines (RKO and HT-29). Stable cell lines were screened with the antibiotic puromycin (MedChemExpress).

For Akt inhibition or activation, 8μM of MK-2206 (HY-10358, MedChemExpress) or 10μg/ml SC79 (HY-18749, MedChemExpress) were used to treat RKO and HT-29 cells for 48 hours, respectively.

### Reverse transcription real-time quantitative polymerase chain reaction (RT‒qPCR)

RT-qPCR was performed according to the previously described method 15. The primer sequences were as follows: PLCG2-Forward: CGAGGCGATGTGGATGTCAA; PLCG2-Reverse: AGTGCCGAGTCCATTTCTGG; ACTB-Forward: GGATGCAGAAGGAGATCACTG; ACTB-Reverse: CGATCCACACGGAGTACTTG.

### Western blotting

The experiments were conducted following the methods previously described in the literature or prior research from our team 16. Briefly, total protein was extracted using RIPA buffer (R0010, Solarbio^®^). Protein concentration was determined with BCA method or Lowry method. The separated proteins were transferred from the SDS-PAGE gels to polyethylene membranes or polyacrylamide membranes. Blocking of non-specific binding sites was performed on the transferred membrane. Subsequently, the membrane was incubated with specific primary antibodies in an appropriate antibody incubation buffer, usually overnight at 4℃. Finally, the target proteins were detected by conjugation with specific secondary antibodies. The antibodies used in this study were listed in Supplementary Table 1.

### Cell immunofluorescence (IF) and immunohistochemistry (IHC)

The cell immunofluorescence experiments were performed according to the previously described method 17. Hyalinization and permeabilization on fixed cells were performed to enhance antibody entry and reduce non-specific background signals. Blocking of non-specific binding sites was performed on both hyalinized and permeabilized cells to inhibit non-specific binding. Subsequently, cells were incubated with specific primary antibodies in a blocking solution. After incubation with the primary antibody, adequate washing was performed, and cells were incubated with specific secondary antibodies (e.g. fluorescently labelled anti-rabbit IgG or anti-mouse IgG) to detect the location and expression of specific antigens. After staining, the nucleus was stained with DAPI. The stained cells were placed under a fluorescence microscope for observation, and the fluorescence signals of specific staining were obtained with the appropriate filter selector. The antibodies and reagents used in this study were listed in Supplementary Table 1.

The IHC experiments were performed according to the previously described method 18. Briefly, appropriate pre-treatment of the tissue involving degreasing, de-hydration, and decalcification was performed to ensure sample integrity and optimal antigen display. Tissue fixation was performed with 4% formaldehyde, and the fixed tissue samples were sectioned. After sectioning, the samples might need to undergo pre-treatment, such as degreasing, deparaffinization, or re-stretching steps, to improve the display of antigens. The sections were subjected to antigen repair to restore the antigenic structure in the sample and to improve the efficiency of antibody binding. Blocking of non-specific binding sites was performed on the antigenically repaired sections to reduce non-specific binding. Subsequently, specific primary antibodies were added to the sections and incubated at the appropriate temperature. After incubation of the primary antibody, adequate washing was carried out and specific secondary antibody suitable for the primary antibody was added. The IHC images were processed with Image-Pro^®^ Plus V6.0 and 3DHISTECH QuantCenter V2.1 software. The specific procedures were described below: (1) Image acquisition: We acquired images of the CRC tissue microarrays using a high-resolution scanning device and 3DHISTECH QuantCenter V2.1 software to ensure that the image quality met the analysis requirements for subsequent quantitative analysis; (2) Image pre-processing: In Image-Pro® Plus V6.0, the necessary pre-processing of the image was performed, including adjusting the contrast and brightness to enhance the PLCG2 staining signal while reducing background noise; (3) Color threshold setting: Based on the staining properties of PLCG2, the specific color threshold was set in the software to distinguish PLCG2 positive expression regions from negative backgrounds; (4) Region selection: Using the region selection tool of Image-Pro® Plus V6.0, the area of interest (AOI) was precisely defined, excluding non-tumor tissue and areas of uneven staining; (5) Quantitative analysis: the software automatically calculated the intensity of PLCG2 staining and the percentage of positive cells in each AOI. The average optical density (AOD) was calculated using the formula: AOD = integrated optical density (IOD) / area. The antibodies used in this study were listed in Supplementary Table 1.

### Culture of organoids derived from CRC patients

Tumor tissues as well as paired para-carcinoma tissue of CRC patients were collected. The basic information, past medical history, treatment history, relevant test parameters, clinicopathological parameters and prognosis of the patients were recorded in detail. Fresh tissues were washed several times with antibiotic-containing PBS, digested enzymatically to approximate single-cell morphology, and washed repeatedly with antibiotic-containing DMEM/F12 medium. Then after the erythrocyte lysis step, the cells were mixed with Matrigel (BD Biosciences), according to the procedure of 3,000-10,000 cells per 5-10μL. After the Matrigel was cured, the organoid medium (WM-H-03, OuMel, China) were added to the wells and mixed with the cells.

### *In vitro* assays for cell proliferation, invasion and metastasis

#### CCK-8 assay

RKO and HT-29 cell lines were seeded separately into 96-well plates at approximately 3000 cells per well. CCK-8 reagent (MA0218, MeilunBio^®^) at the concentration of 10% was added to each well, and the culture dish was gently shaken to ensure uniform distribution of the reagent. The CCK-8 reagent was incubated with the cells for 2h, and the wavelength of 450 nm was used to measure the absorbance of each well with a microplate reader (ThermoFisher, USA). The proliferation detection time points were 6h, 24h, 48h, 72h and 96h. Five biological replicates and three technical replicates were used in each group.

#### Colony formation assay

Cells were homogeneously inoculated into 6-well plates at 1000 per well for sufficient culture cycles, usually 10-14 days, so that individual cells could grow and form clones visible to the naked eye. Wash twice with PBS, fix with formaldehyde, stain with 0.5% crystal violet, wash repeatedly with PBS and air dry. Photographs were taken and counted. Each group contained three biological replicates and three technical replicates.

#### Wound healing assay

A 6-well plate was used, and the appropriate number of cells were inoculated evenly in it. When the cells were fully grown, they were washed with PBS. Form the wound zone by drawing a straight line through the cell monolayer with the 10-microliter gun tip. Ensure that the wound length was consistent and record the location of the trauma. The culture dish was washed with PBS to remove the detached cell debris, followed by the addition of serum-free DMEM basal medium. The healing of the wound zone was observed and recorded at 0 and 24 hours. The migrated area of the wound zone was analyzed with Image J software. Each group contained three biological replicates and three technical replicates.

#### Transwell assay

First, cells were pre-treated in serum-free medium for 24h. Then a total of 5×10^4^ cells were added into serum-free basal medium and seeded onto the upper chamber of a pre-sterilized transwell plate (24-well insert, 8μm pore size) (Corning Costar, Cambridge, MA, USA). The lower chamber was filled with a complete medium containing 10% FBS, and the plate was placed back into the incubator at 37°C with 5% CO_2_ for 24 hours. At the end of the culture, cells in the upper chamber and cells that could not pass through the culture membrane were gently removed. Then, the transwell plates were fixed and stained with 0.5% crystal violet, and cells passing through the transwell membrane were observed with an inverted microscope (IX71; Olympus, Tokyo, Japan). Five fields of view per chamber were randomly selected, and the mean cell number was calculated. For invasion research, 50μL of diluted matrix gel (the proportion of Matrigel: DMEM medium = 1:5) was added to the upper layer of the transwell chambers. Three biological replicates and three technical replicates were used for each group.

#### Cell cycle and apoptosis assay

For cell cycle analysis, cells were digested with 0.25% trypsin and collected by centrifugation after reaching approximately 70-80% fusion. Cells were resuspended with pre-cooled PBS, pre-cooled anhydrous ethanol was slowly added, gently blown to mix, and fixed overnight at 4°C. The fixed cells were taken out and equilibrated to room temperature. Resuspend with pre-cooled PBS and centrifuge, and aspirate the supernatant. Cells were resuspended by adding 1 mL PBS and transferred to the assay tube, centrifuged and the supernatant removed. Cells were resuspended by adding 300μL PI/ RNase staining (550825, BD Biosciences, USA) and incubated at 37°C for 30 min protected from light. The stained cell suspension was analyzed by flow cytometry (BD FACSCalibur, USA). The collected data were analyzed by CellQuest Pro software. Each group contained five biological replicates and three technical replicates.

For apoptosis assay, after the cells reached about 80-90% fusion, the old culture solution was retained into 15 ml centrifuge tubes. Cells were collected by trypsin digestion, mixed with the old culture medium, centrifuged and the supernatant removed. After washing the cells twice, they were resuspended with 100μL 1×Binding Buffer (556454, BD Biosciences, USA) and transferred to the detection tubes. 3μL Annexin V-APC (550474, BD Biosciences, USA) and 5μL 7-AAD (559925, BD Biosciences, USA) were added to each sample tube. After adding the dye, mix gently to fully bind the cells to the dye and incubate for 15 min at room temperature away from light. The sample system was expanded by adding 200μL 1×Binding Buffer and the stained cell suspension was analyzed by flow cytometry (BD FACSCalibur, USA). The collected data were analyzed by CellQuest Pro software. Each group contained five biological replicates and three technical replicates.

### Animal experiments

Four-week-old male BALB/c nude mice and male C57BL/6 mice for this study were obtained from PHENOTEK Biotechnology (Shanghai) Co., LTD.

#### Subcutaneous xenograft model in nude mice

Male nude mice (BALB/c nu/nu) were subcutaneously injected with lentivirus-transfected RKO cell suspension at the concentration of 5×10^6^ cells/mL in a volume of 100μL on the back of the nude mice. To ensure effective cell implantation, the inoculated nude mice were placed in the constant temperature feeding box for recovery for at least 2h. The weight, tumor volume and general health of the nude mice were regularly observed every 4 days, and any abnormalities were promptly recorded and reported. The formula for evaluating tumor volume was volume = (length × width^2^)/2.

#### Lung metastasis model in nude mice via tail vein injection

Lentivirus-transfected and luciferase-labelled HT-29 cells were digested by trypsin, and single-cell suspension was collected prior to injection and adjusted to the final concentration of 1×10^6^ cells/mL. Under sterile conditions, each male nude mouse was injected with 100μL cell suspension via the tail vein. After 4 weeks, nude mice were imaged fluorescently by the small animal live imaging system to assess the number and size of lung metastatic nodules. Finally, lung tissues were removed through the sterile surgical procedure and analyzed by histopathology to confirm the presence of metastatic tumors.

#### *In vivo* treatment model of subcutaneous tumor in C57BL/6 mice

Lentivirus-transfected RKO cell concentration was adjusted to 5×10^6^ cells/mL, followed by injection of 100μL cell suspension under the back of each mouse under aseptic conditions to establish the subcutaneous tumor model. After injection, body weight monitoring and tumor observation were performed once a day, and their health status was assessed. When the volume of the subcutaneous tumor reached approximately 100mm^3^, the mice were randomly distributed into oenc-PLCG2 group, oe-PLCG2 group and oe-PLCG2+MK2206 group with 6 mice in each group. The oe-PLCG2+MK2206 group was treated with MK2206, and the oenc-PLCG2 group and oe-PLCG2 group were treated with equal volumes of PBS solution in the same administration mode and administration time. MK2206 (oral gavage, 150mg/kg in 200μL PBS) was administered every three days for three weeks. Finally, the tumors were removed for subsequent IHC experiments and molecular biology testing.

Lentivirus-transfected MC38 cells were digested by trypsin and single-cell suspension was collected, and the cell concentration was adjusted to 2×10^6^ cells/mL, followed by injection of 100μL cell suspension under the back of each mouse under aseptic conditions to establish the subcutaneous tumor model. After injection, body weight monitoring and tumor observation were performed once a day, and their health status was assessed. When the volume of the subcutaneous tumor was approximately 100mm^3^, the mice were randomly divided into shnc-PLCG2+IgG group, shnc-PLCG2+anti-PD-1 group, sh-PLCG2+IgG group and sh-PLCG2+anti-PD-1 group, with 6 mice in each group. Mice in each group were treated with InVivoMAb anti-mouse PD-1 (BE0146, BioXcell) and IgG isotype control (BE0089, BioXcell), and the *in vivo* treatment monoclonal antibodies were administered via intraperitoneal injection (100μg in 100μL D-PBS buffer per mouse) every three days for up to three weeks. The body weight, tumor growth and general health of the mice were regularly observed, and any abnormalities were promptly recorded and reported. Finally, the tumors were removed for subsequent IHC experiments and molecular biology testing. A schematic diagram of the specific experimental protocol was shown in Figure 10A.

### High-throughput transcriptome sequencing

High-throughput transcriptome sequencing of RKO cells stably transfected with oenc-PLCG2 lentivirus (n=5) and oe-PLCG2 lentivirus (n=5) was performed with the aim of mining the biological function and signaling pathways regulated by PLCG2 in CRC. High-throughput transcriptome sequencing was performed by ApexBio Technology LLC. (Shanghai China) using the Illumina NovaSeq 6000 sequencing platform (Paired end150). TrimGalore filtered the raw data by invoking the Cutadapt tool to discard sequences containing adapters and low-quality bases. The obtained sequencing data was aligned with the hg38 (GRCh38) human genome using HISAT2 (v2.1.0), and a bam file was generated by aligning the sequences of the reference genome. The transcripts were assembled and counted for gene abundance expression using StringTie, and TPM was used as a normalized value. Differentially expressed genes (DEGs) were identified by edgeR with the differential gene screening threshold of log_2_|foldchange|>1 and *P* adjust-value<0.05.

### Flow cytometry analysis of tumor tissues

After the sacrifice of the C57BL/6 mice, their subcutaneous tumor tissue was rapidly removed and placed in pre-cooled PBS. Tumor samples were separated into small pieces under aseptic conditions and tissue digestive enzymes were utilized in order to prepare them into single cell suspensions. To remove cell debris and non-cell components, the cell suspension was filtered through the 70μm cell filter. The single cell suspension was split into two parts. Three-quarters of the single-cell suspension was resuspended and non-immune cells were removed. Cells were stimulated using Leukocyte Activation Cocktail (550583, BD biosciences, San Jose, CA, USA) and incubated at 37°C for 4-6 h according to the manufacturer's instructions, followed by staining with LIVE/DEAD Fixable Violet DEAD Cell Staining Kit (ThermoFisher Scientific) for 40 min on ice protected from light. Cells were subsequently blocked with TruStain FcX™ (BioLegend, San Diego, CA, USA; anti-mouse CD16/32) antibody and stained for 30 min on ice using flow cytometry antibodies to detect the expression of CD45, CD3, and CD8 on the cell surface. The cells were fixed and permeabilized with eBioscience™ FOXP3/Transcription Factor Staining Buffer Set (00-5523-00, ThermoFisher). Cells were subsequently stained in the permeabilized solution for PD-1, IFN-γ, GZMB, TNF-α and PRF1. The remaining one-quarter of the single-cell suspension was stained and separated from live and dead cells using the LIVE/DEAD Fixable Violet DEAD Cell Staining Kit and blocked using TruStain FcX™. Next, flow cytometry antibodies CD326 (EpCAM) and PD-L1 were used to detect PD-L1 expression on the tumor cell surface. Samples and corresponding data were analyzed on the Beckman CytoFLEX LX (Becton Dickinson, Franklin Lakes, NJ, USA) by flow cytometry and Flow Jo software (Becton Dickinson), respectively. The flow cytometry antibodies used in this study were listed in Supplementary Table 1.

### Statistical analysis

Statistical analyses were performed using R Software (Version 4.1.0) and GraphPad Prism software (Version 8.0). The measurement data were described as means ± standard deviation (SD). Comparisons of the differences in continuous variables between the two groups with or without normal distribution were conducted using unpaired Student's *t*-test or Wilcoxon rank-sum test. One-way ANOVA was used for comparisons between three or more groups. The two-sided* P*-value<0.05 was considered statistically significant.

## Results

### Expression profile and protein localization of PLCG2 in CRC

**Figure 1:**
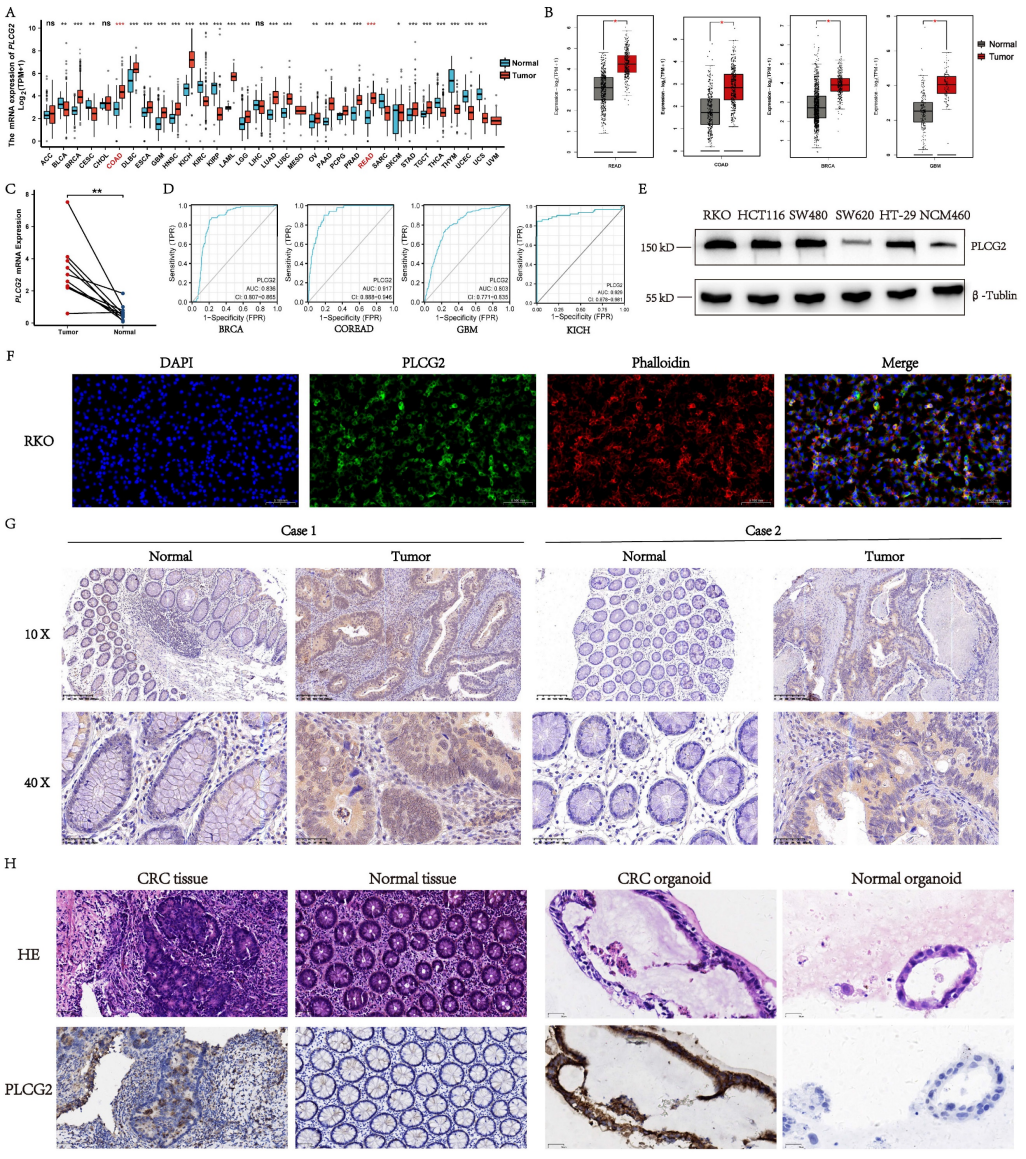
Expression profile and protein localization of PLCG2 in CRC. **A** The mRNA expression of PLCG2 in pan-cancer. Data were obtained from TCGA official website. **B** The mRNA expression of PLCG2 in the TCGA and GTEx integrated cohorts of the GEPIA2 database. Data were obtained from GEPIA2 official website. **C** qRT-PCR results of matched CRC tumor tissue (n=10) and normal tissue (n=10) from our medical center. **D** The diagnostic performance of PLCG2 in BRCA, COREAD, GBM and KICH. **E** The PLCG2 protein expression in CRC cell lines (RKO, HCT116, SW480, SW620 and HT-29) and normal colon epithelial cell line (NCM460). **F** The IF of RKO cell line. Scale bar=0.100mm. **G** The protein expression of PLCG2 in CRC clinical samples was evaluated by IHC (n=76). Scale bar=200μm (10X). Scale bar=50μm (40X). **H** The protein expression of PLCG2 in CRC organoids was evaluated by IHC (n=3). Scale bar=50μm. Data were presented as mean±SD. ns no statistical significance, *P<0.05, **P<0.01, ***P<0.001, ****P<0.0001. PLCG2 phospholipase Cγ2, ACC adrenocortical carcinoma, BLCA bladder urothelial carcinoma, BRCA breast invasive carcinoma, CESC cervical squamous cell carcinoma and endocervical adenocarcinoma, CHOL cholangiocarcinoma, COAD colon adenocarcinoma, DLBC lymphoid neoplasm diffuse large B-cell lymphoma, ESCA esophageal carcinoma, GBM glioblastoma multiforme, HNSC head and neck squamous cell carcinoma, KICH kidney chromophobe, KIRC kidney renal clear cell carcinoma, KIRP kidney renal papillary cell carcinoma, LAML acute myeloid leukemia, LGG brain lower grade glioma, LIHC liver hepatocellular carcinoma, LUAD lung adenocarcinoma, LUSC lung squamous cell carcinoma, MESO mesothelioma, OV ovarian serous cystadenocarcinoma, PAAD pancreatic adenocarcinoma, PCPG pheochromocytoma and paraganglioma, PRAD prostate adenocarcinoma, READ rectum adenocarcinoma, SARC sarcoma, SKCM skin cutaneous melanoma, STAD stomach adenocarcinoma, TGCT testicular germ cell tumors, THCA thyroid carcinoma, THYM thymoma, UCEC uterine corpus endometrial carcinoma, UCS uterine carcinosarcoma, UVM uveal melanoma.

To explore the mRNA expression profile of PLCG2 in pan-cancer, we examined the mRNA expression differences of PLCG2 between tumor and normal tissues in TCGA public database. As illustrated in Figure 1A, *PLCG2* mRNA expression in tumor tissues was significantly different from normal tissues in pan-cancer. In the TCGA-COAD and TCGA-READ cohorts, a higher *PLCG2* mRNA expression was observed in tumor tissues. Similarly, based on the integrated cohorts of TCGA and GTEx in the GEPIA2 database, we found significantly increased mRNA expression of *PLCG2* in tumor tissues in READ, COAD, BRCA, and GBM (Figure 1B). We further validated PLCG*2* mRNA expression in CRC by collecting 10 pairs of normal and tumor tissues from our center. With the use of qRT-PCR, *PLCG2* mRNA expression was found consistent with that of the previous cohorts (Figure 1C). An excellent diagnostic performance of PLCG2 was demonstrated in BRCA (0.836, 95%CI: 0.807-0.865), COREAD (0.917, 95%CI:0.888-0.946), GBM (0.803, 95%CI:0.771-0.835), and KICH (0.929, 95%CI:0.878-0.981) respectively, as shown in Figure 1D. We then explored the expression of PLCG2 in CRC at the protein level. Except for SW620, the protein expression of PLCG2 was significantly higher in CRC cell lines (RKO, HCT116, SW480, and HT29) compared to the normal colonic epithelial cell line (NCM460) (Figure 1E). In terms of the protein localization of PLCG2 in CRC cells, the multiplex immunohistochemistry (mIHC) results of the RKO cell line showed that PLCG2 was mainly distributed in the cell membrane and cytoplasm, which represented the structural basis for the function of cell signal transduction proteins (Figure 1F). IHC experiments were carried out for further validation, of which the results indicated that tumor tissues exhibited significantly higher PLCG2 protein expression than the matched normal ones (Figure 1G). The organoids derived from pathological tissues of CRC patients also confirmed that PLCG2 protein expression in CRC tissues and tumor organoids was significantly higher compared to matched normal tissues and normal intestinal epithelial organoids (Figure 1H). Based on the results above, we believed that PLCG2 might have an indispensable role in CRC tumorigenesis and progression, given its significantly high expression in CRC.

### Mutational landscape analysis in CRC between the high and low PLCG2 expression groups

To gain insights into the diversities of the mutational landscape between the two groups with high and low PLCG2 expression in CRC, we analyzed simple nucleotide variant data from the TCGA cohort. In the TCGA cohort, we classified CRC patients into high (high PLCG2 expression) and low-risk (low PLCG2 expression) groups using the median PLCG2 expression value. Firstly, we investigated the frequency and type of PLCG2 mutations in pan-cancer datasets, and the results showed that PLCG2 was most pronounced in melanoma, with a mutation frequency of more than 10%. Unsurprisingly, PLCG2 mutation frequency in CRC was also high, which was more than 6%, and the two main types of mutation were "mutation" and "deep deletion" (Figure S1A). In the PLCG2 unaltered group, the molecular sub-types of CRC patients were predominantly CIN, whereas the CRC patients in the PLCG2 altered group predominantly had the molecular sub-type of MSI. This result suggested that mutations in PLCG2 may be associated with the molecular typing of CRC, which laid the groundwork for comprehending the molecular mechanism of PLCG2 heterogeneity in CRC (Figure S1B). Survival analyses indicated that CRC patients carrying PLCG2 mutations were associated with significantly shorter overall survival than those without mutations (Figure S1C). Figure S1D illustrated the mutations on different protein structural domains of PLCG2, with more apparent mutation frequencies on PI-PLC-X and SH2 structural domains. Subsequently, by analyzing the changes of somatic mutations in two different PLCG2 expression groups, we discovered that the high PLCG2 expression group had APC (73%), TP53 (59%), TTN (46%), KRAS (44%), SYNE1 (32%), and PIK3CA (31%) as the top six candidates exhibiting the highest mutation frequencies (Figure S1E). In contrast, the low PLCG2 expression group had APC (69%), TP53 (50%), TTN (47%), KRAS (42%), PIK3CA (29%), and MUC16 (25%) manifesting the highest mutation frequencies (Figure S1F). The development of CRC was a multi-gene process, and APC, TP53, TTN, KRAS, and PIK3CA were well-known genes closely related to CRC. APC was an oncogene in CRC, and most of its mutations occurred in the early stages. Encoded by the APC gene, the APC protein acted as an effector of the Wnt signaling pathway to regulate β-catenin expression. TP53 was one of the most important oncogenes, and its mutations were associated with more than half of all tumors. p53 was a transcription factor, and many of these target genes were involved in apoptosis or cell cycle regulation. The analysis results illustrated that there was a close connection between the high expression of PLCG2 and the high frequency of mutations in the tumor suppressor genes APC and TP53, suggesting that the high expression of PLCG2 may affect the somatic mutations in CRC patients and promote the tumorigenesis and progression of CRC. The occurrence of gene mutations was highly random, but the combination of mutated genes retained after clonal evolution that culminated in a tumor should exert some synergistic effects. At the same time, specific mutant genes in different sub-clones had certain mutual exclusion. The analysis of mutual exclusivity and co-occurrence of mutant genes was used to distinguish the co-occurrence and mutually exclusive mutant genes, which helped to identify mutant genes specific to the tumor sub-clones and define the tumor sub-types. As a result, it was possible to differentiate important functional somatic mutations arising from similar tumors, which provided crucial references to locate the driver genes. In the group with high PLCG2 expression, APC was mutually exclusive with KMT2B, ZFHX4, OBSCN, FAT4, and PIK3CA, while TP53 was mutually exclusive with PCLO, OBSCN, and PIK3CA. Moreover, TTN co-occurred with USH2A, KMT2B, ADGRV1, and FAT3 (Figure S1E). In the group with low PLCG2 expression, APC co-occurred with KRAS and TP53, whereas TP53 was mutually exclusive with ADGRV1, ANK3, and MUC16. In addition, TTN co-occurred with SYNE2, ADGRV1, NEB, FAT3, and MUC16 (Figure S1F). Further, RTK-RAS, WNT, NOTCH, and Hippo signaling pathways were significantly enriched in PLCG2 high-expression group (Figure S1G) and PLCG2 low-expression group (Figure S1H). Overall, mutational landscape analysis of CRC patients based on PLCG2 expression suggested the important role of PLCG2 in the tumorigenesis and progression of CRC.

### PLCG2 expression was correlated with prognosis and clinicopathological features in CRC patients

In the TCGA-COREAD cohort, we observed that CRC patients in the high-risk group had significantly worse prognosis. The survival status plot indicated that as the expression of PLCG2 increased, the survival time of CRC patients became shorter, and the survival status was poorer (Figure S2A). The same conclusion was made in the GSE39582 cohort as well (Figure S2B). Figure S2C presented representative IHC images of high and low PLCG2 expression of CRC patients from the Ruijin cohort at our medical center. In the Ruijin cohort, it was further validated that high expression of PLCG2 led to poor prognosis in CRC patients (Figure S2D). The ROC curves for the TCGA-COREAD, GSE39582, and Ruijin cohorts all demonstrated that PLCG2 maintained a better prognostic accuracy for CRC patients. While the AUC values did not show a decreasing trend with increasing survival time, PLCG2 remained a valuable prognostic marker with satisfactory performance across the predicted time points (Figure S2E).

To uncover the relationship between PLCG2 expression and clinicopathological features of CRC patients, we employed a complex heatmap to depict the pattern of clinicopathological features (Status, Age, Gender, pT, pN, pM, Stage, Location) (Figure S3A) in two different PLCG2 expression groups. In the TCGA-COREAD (Figure S3B) and GSE39582 cohorts (Figure S3C), higher pT stage, pM stage, TNM stage, and right-hemi tumors were significantly associated with higher PLCG2 expression. Similarly, the Ruijin cohort also showed that CRC patients with high expression of PLCG2 might present with higher pT stage, pM stage, TNM stage, and right-hemi tumors (Figure S3D). The association between PLCG2 expression and the clinicopathological characteristics in the Ruijin cohort was presented in Table 1.

### PLCG2 shaped the heterogeneity of CRC tumor microenvironment as revealed by single-cell and spatial transcriptome

**Figure 2:**
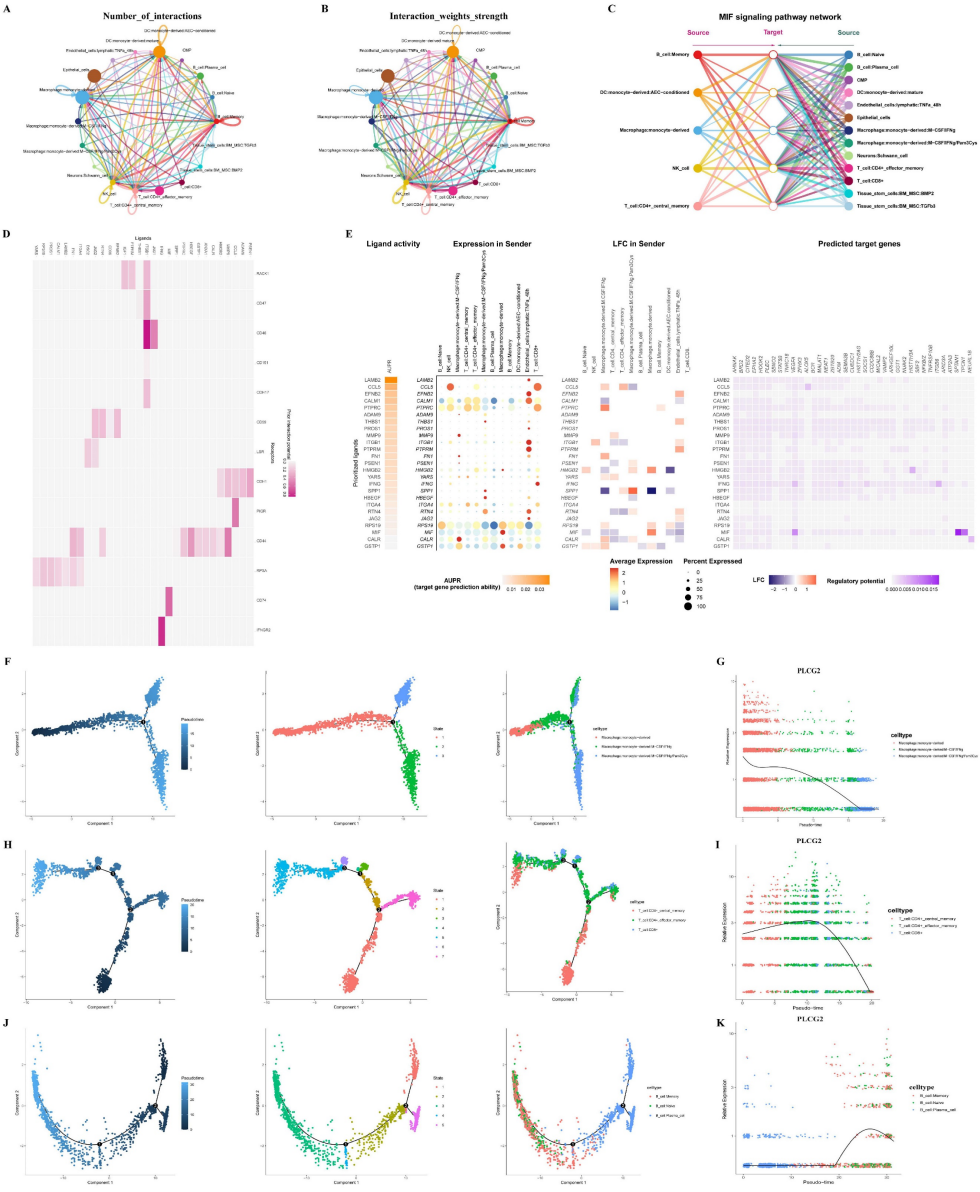
Cell communication and pseudo-time analysis for PLCG2 in single-cell transcriptome. **A** The number of interactions in cell communication among different cell subpopulations. **B** The weights of interactions in cell communication among different cell subpopulations. **C** The "MIF signaling pathway network" in cell communication. **D** The probability of prior interactions between the ligands in the sender cells and the receptors in the epithelial cells. **E** The ligand expression and activity in the sender cells and the probability of prior interactions with predicted target genes in the epithelial cells. **F** The cell trajectory analysis of macrophages. **G** The changes of PLCG2 expression with pseudo-time in macrophages. **H** The cell trajectory analysis of T cells. **I** The changes of PLCG2 expression with pseudo-time in T cells. **J** The cell trajectory analysis of B cells. **K** The changes of PLCG2 expression with pseudo-time in B cells. Data were presented as mean±SD. ns no statistical significance, *P<0.05, **P<0.01, ***P<0.001, ****P<0.0001. t-SNE t-Distributed Stochastic Neighbor Embedding, PLCG2 phospholipase Cγ2, MIF macrophage migration inhibitory factor.

Single-cell transcriptional profiles of six CRC tissue specimens were revealed to investigate the important role of PLCG2 in the CRC tumor microenvironment. Figure S4A demonstrated single-cell t-SNE clustering according to patient ID number, and the results showed that batch effects and inter-individual variation of samples were minor after rigorous quality control. 33,538 cells were clustered into 23 cell subpopulations (Figure S4B). The annotation of cell subpopulations was performed with the "SingleR" R package (Figure S4C). Figure S4D demonstrated the expression of PLCG2 in various cell subpopulations. We used the "CellChat" R package to explore cell communication among different cell subpopulations in the CRC microenvironment, including the number of interactions (Figure 2A) and the weights of interactions (Figure 2B). Among them, the "MIF signaling pathway network" was an important cell communication signaling pathway in the CRC microenvironment (Figure 2C). To further explore the role of PLCG2 in tumor cells in cell communication with other cell subpopulations, we investigated cell communication between epithelial cells expressing high and low PLCG2 as the receptor cells and other cell subpopulations using the "NicheNet" R package. Figure 2D presented the probability of prior interactions between the ligands in the sender cells and the receptors in the epithelial cells. The ligand expression and activity in the sender cells and the probability of prior interactions with predicted target genes in the epithelial cells were presented in Figure 2E. Given that macrophages, T cells, and B cells were important immune cells in the CRC microenvironment, we carried out cell trajectory analysis to explore the important role of PLCG2 in the development and differentiation of immune cells. Macrophage subpopulations were characterized into three states (Figure 2F), with PLCG2 expression gradually decreasing with pseudo-time (Figure 2G). T cell subpopulations were divided into 7 states (Figure 2H), and PLCG2 expression showed a small increase followed by a large decrease with pseudo-time (Figure 2I). B cell subpopulations were categorized into 5 states (Figure 2J), of which PLCG2 expression showed a large increase followed by a small decrease with pseudo-time (Figure 2K). The above results revealed the expression of PLCG2 in the CRC microenvironment and its important role in cell communication and the development and differentiation of immune cells.

To understand the spatial distribution of PLCG2 in CRC tissues, we carried out spatial transcriptome sequencing (M1002). The hematoxylin-eosin (HE) staining of the CRC tissue specimen was presented in Figure S5A. The spots of the spatial transcriptome were divided into seven regional subgroups in total, and the regional subgroups were annotated according to the classical cell markers (Figure S5B). Figure S5C showed the regional subgroups based on t-SNE downscaling and clustering. The PLCG2 expression in the spatial distribution of tissues and regional subgroups was shown in Figure S5D, E, F, and G.

### PLCG2 promoted the growth and proliferation of CRC cells and induced the cell cycle from G0+G1 phase to S phase

**Figure 3:**
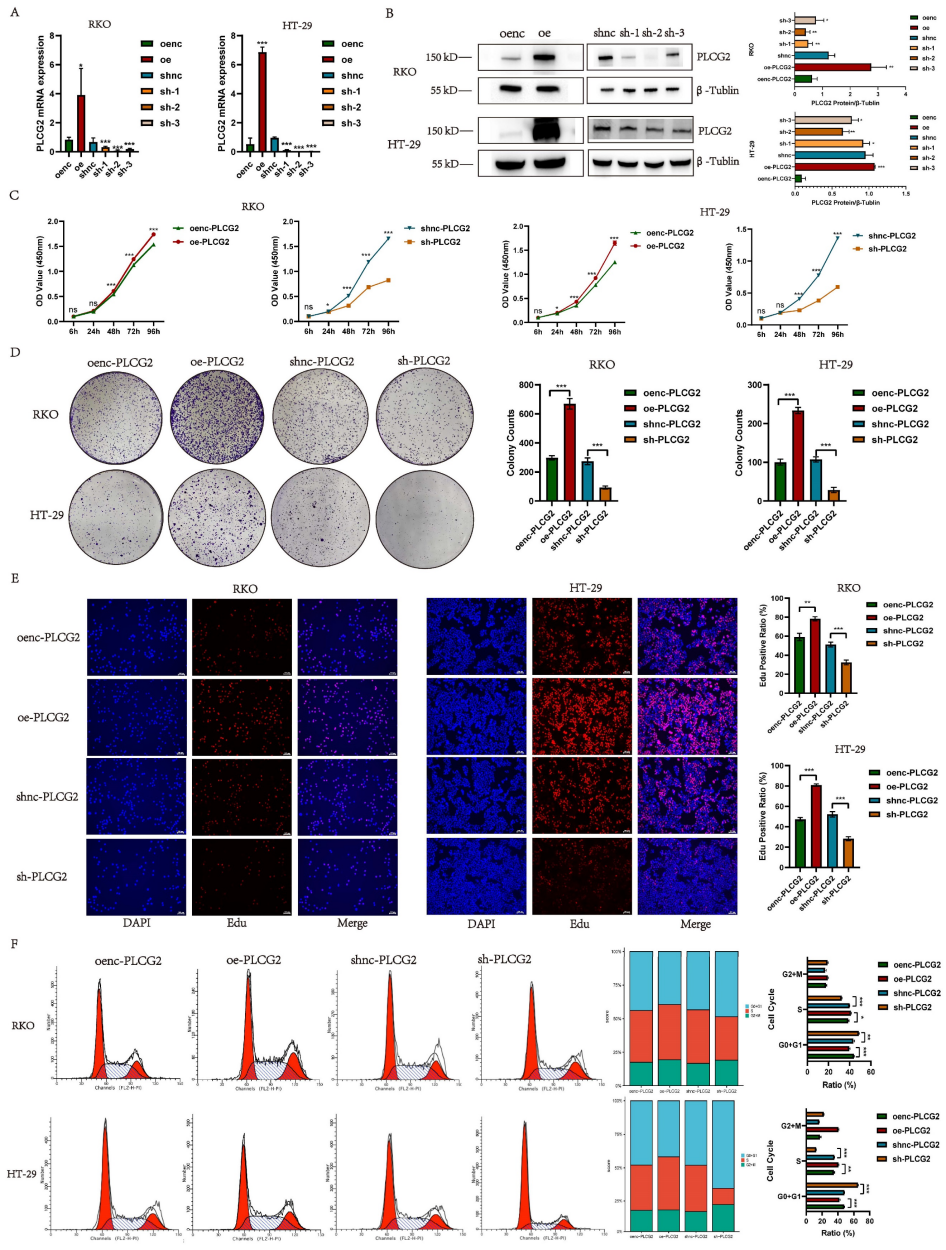
PLCG2 promoted the growth and proliferation of CRC cells and induced the cell cycle from G0+G1 phase to S phase. **A** The transfection efficiency of lentivirus was verified by qRT-PCR (n=5). **B** The transfection efficiency of lentivirus was verified by western blotting (n=3). **C** CCK-8 assay (n=5). **D** Colony formation assay (n=3). **E** EdU assay (n=3). Scale bar=100μm. **F** Cell cycle detection by flow cytometry (n=5). Data were presented as mean±SD. ns no statistical significance, *P<0.05, **P<0.01, ***P<0.001, ****P<0.0001. oenc overexpression negative control, oe overexpression, shnc short hairpin RNA negative control, sh short hairpin RNA, Edu 5-ethynyl-2'-deoxyuridine.

To unveil the biological function of PLCG2 in CRC cells, we constructed PLCG2-overexpressing and knockdown CRC cell lines transfected with lentiviral vectors (RKO and HT-29). qRT-PCR and western blotting were employed to validate the transfection efficiency. The qRT-PCR results showed that cells transfected with PLCG2-overexpressing lentivirus had significantly higher mRNA expression than the control group, whereas cells transfected with three PLCG2-knockdown lentiviruses showed significantly lower mRNA expression than the control group (Figure 3A). Western blotting showed that cells transfected with PLCG2-overexpressing lentivirus had significantly increased protein expression than the control group. Conversely, protein expression was significantly lower in cells transfected with PLCG2-knockdown lentivirus compared with the control ones, with the lowest protein expression in cells transfected with sh-2 lentivirus (Figure 3B). Therefore, cells transfected with sh-2 lentivirus were used as PLCG2-knockdown group, which was applied to subsequent *in vivo* and *in vitro* experiments. The results of CCK-8 assay (Figure 3C), colony formation assay (Figure 3D). and EdU assay (Figure 3E) indicated that the growth and proliferation abilities of CRC cells were significantly promoted by PLCG2. PLCG2 also influenced the cell cycle of the tumor cells. It was observed that overexpression of PLCG2 promoted the transition from G0+G1 phase to S-phase, whereas knockdown of PLCG2 resulted in G0+G1 phase arrest and inhibited the transition to S-phase (Figure 3F).

### PLCG2 promoted migration, invasion and EMT of CRC cells

**Figure 4:**
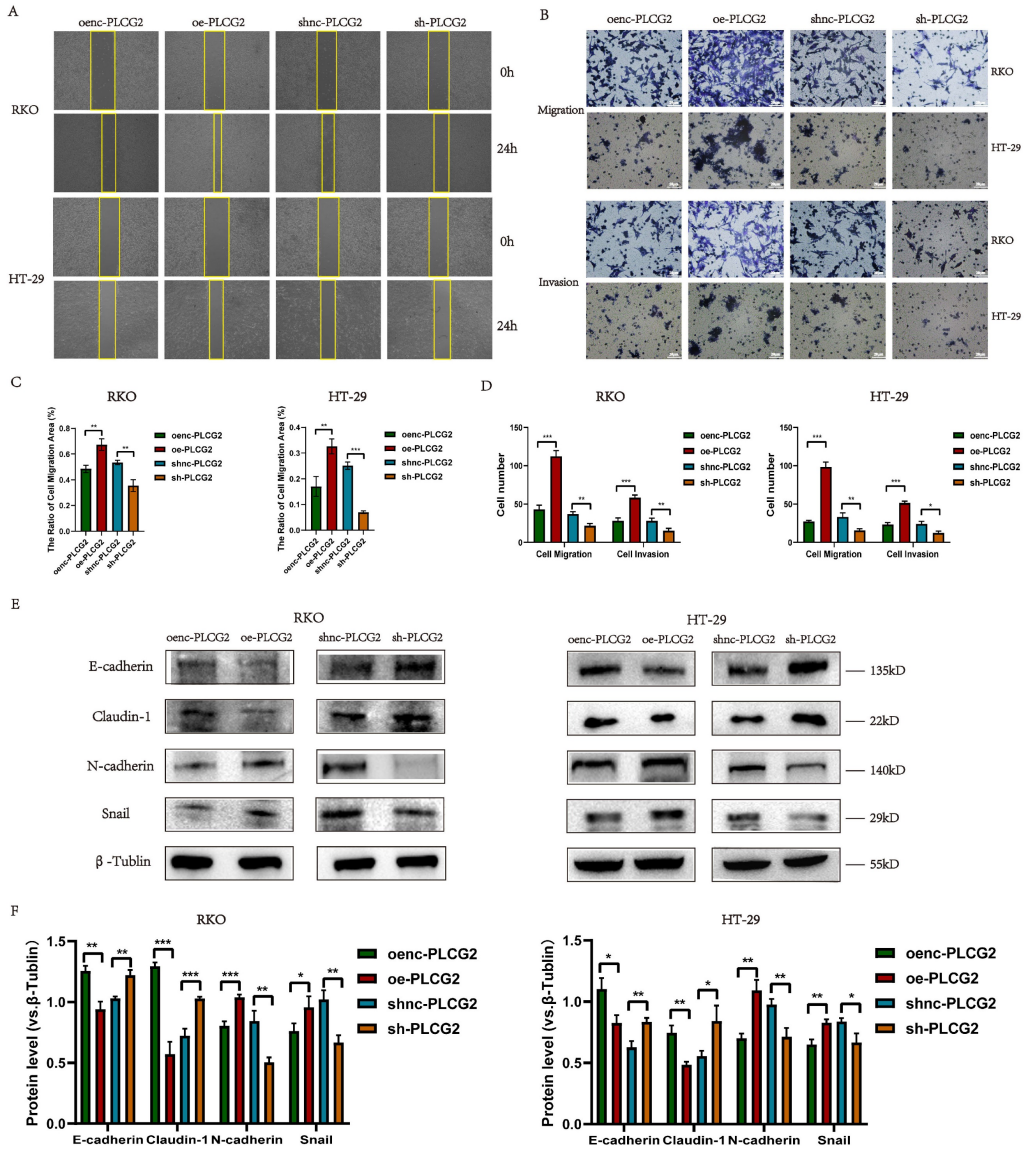
PLCG2 promoted migration, invasion and EMT of CRC cells. **A** Wound healing assay (n=3). **B** Transwell assay (n=3). Scale bar=20μm. **C** The quantitative results of wound healing assay (n=3). **D** The quantitative results of transwell assay (n=3). **E** The protein expression of EMT-related markers was detected by western blotting (n=3). **F** The quantitative results of western blotting (n=3). Data were presented as mean±SD. ns no statistical significance, *P<0.05, **P<0.01, ***P<0.001, ****P<0.0001. oenc overexpression negative control, oe overexpression, shnc short hairpin RNA negative control, sh short hairpin RNA.

We delved into the role of PLCG2 on the metastatic capability of tumor cells by *in vitro* experiments in CRC cells. CRC cell migration was detected by the wound healing assay, which showed that overexpression of PLCG2 significantly promoted the migration, and knockdown of PLCG2 significantly suppressed the migration after 24 h (Figure 4A, C). Transwell assay further demonstrated that overexpression of PLCG2 significantly enhanced CRC cell migration and invasion, while knockdown of PLCG2 showed the reverse effects (Figure 4B, D). EMT, which was highly related to the migration and invasion capability of CRC cells, was characterized by the absence of epithelial cell markers and the up-regulation of mesenchymal cell markers. Hence, we further assessed the effect of PLCG2 on EMT of CRC cells. It was evident that overexpression of PLCG2 significantly downregulated the protein expression of epithelial cell markers (E-cadherin and Claudin-1) and upregulated the protein expression of mesenchymal cell markers (N-cadherin and Snail), and the inhibition of EMT was seen in knockdown of PLCG2 (Figure 4E, F).

### PLCG2 inhibited the apoptosis of tumor cells and the expression of apoptosis-promoting proteins

**Figure 5:**
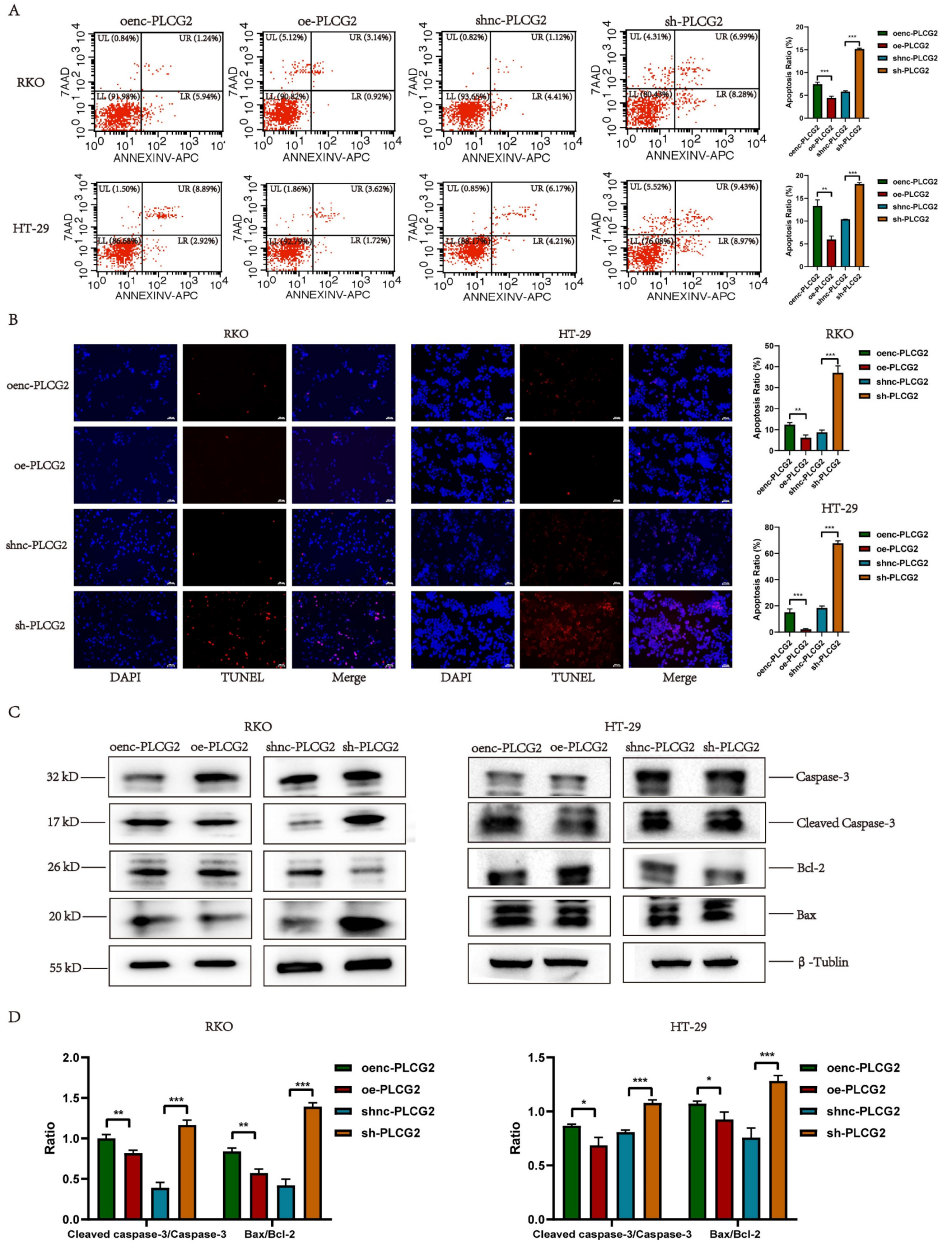
PLCG2 inhibited the apoptosis of tumor cells and the expression of apoptosis-promoting proteins. **A** Apoptosis detection by flow cytometry (n=5). **B** Apoptosis detection by TUNEL staining (n=3). Scale bar=100μm. **C** The protein expression of apoptosis-related markers was detected by western blotting (n=3). **D** The quantitative results of western blotting (n=3). Data were presented as mean±SD. ns no statistical significance, *P<0.05, **P<0.01, ***P<0.001, ****P<0.0001. oenc overexpression negative control, oe overexpression, shnc short hairpin RNA negative control, sh short hairpin RNA, TUNEL TdT-mediated dUTP Nick-End Labeling.

To further investigate whether PLCG2 had an impact on apoptosis in CRC cells, we detected apoptosis in CRC cells with overexpression and knockdown of PLCG2 by flow cytometry and TUNEL staining. The results of flow cytometry (Figure 5A) and TUNEL staining (Figure 5B) showed that overexpression of PLCG2 significantly inhibited apoptosis in CRC cells, and knockdown of PLCG2 promoted apoptosis. Subsequently, we analyzed the expression of apoptosis-related proteins. Overexpression of PLCG2 significantly decreased the ratios of Cleaved Caspase-3/Caspase 3 and Bax/Bcl-2, whereas knockdown of PLCG2 significantly increased the ratios, indicating that overexpression of PLCG2 increased the expression of the apoptosis-inhibiting related proteins and decreased the expression of apoptosis-promoting related proteins, thus suppressed the apoptosis of CRC cells (Figure 5C, D).

### PLCG2 promoted tumor growth, inhibited apoptosis and facilitated lung metastasis *in vivo*

**Figure 6:**
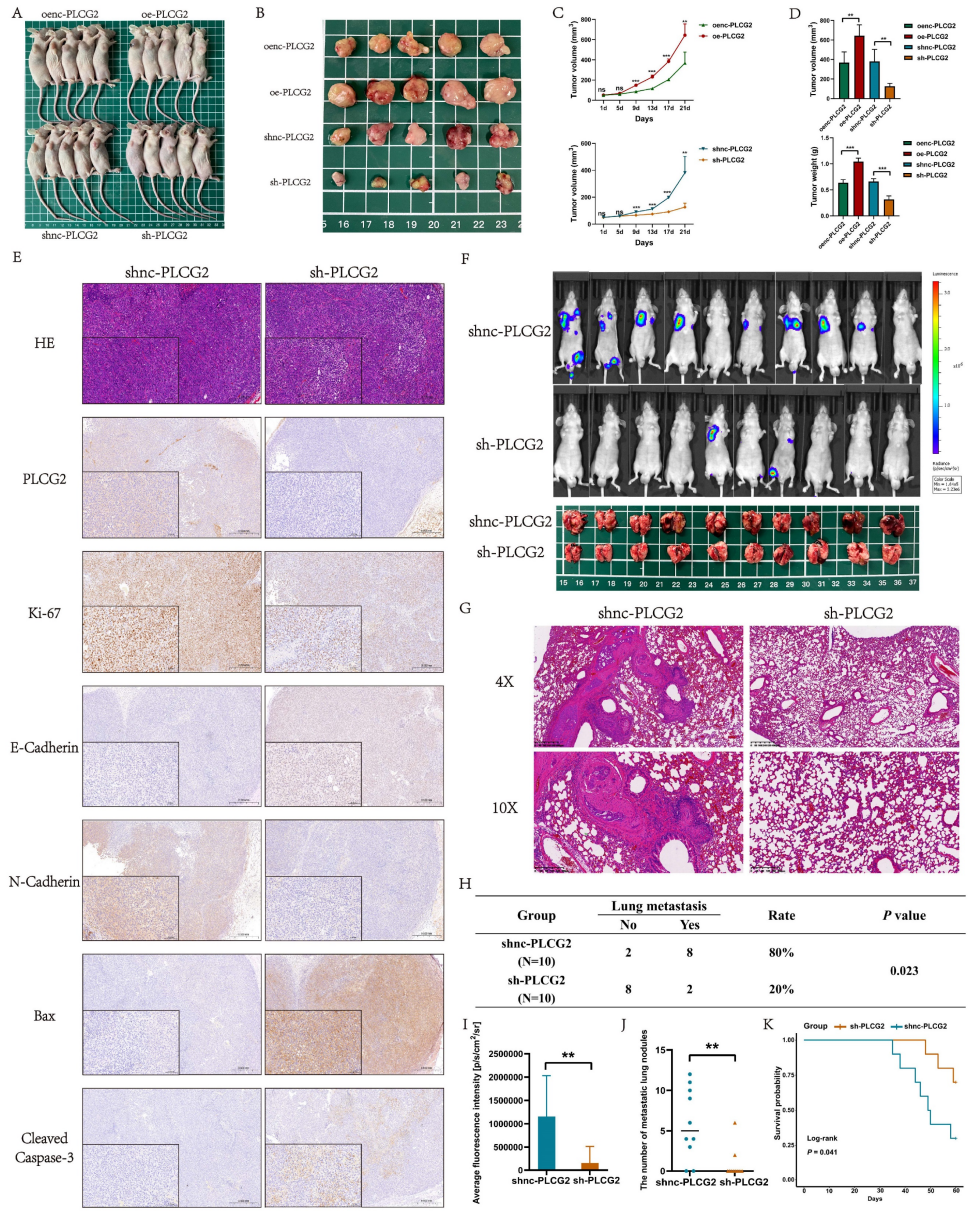
PLCG2 promoted tumor growth, inhibited apoptosis and facilitated lung metastasis *in vivo*. **A** Xenografted nude mice at the end of the experiment (n=5). **B** Subcutaneous tumors at the end of the experiment (n=5). **C** Growth curves of subcutaneous tumor (n=5). **D** Subcutaneous tumor volume and tumor weight (n=5). **E** Representative IHC images of subcutaneous tumors (n=5). **F** Fluorescence imaging *in vivo* and macroscopic observation of lung metastasis at the end of the experiment (n=10). **G** H&E staining of lung tissue (n=10). **H** Comparison of incidence of lung metastasis between shnc-PLCG2 and sh-PLCG2 groups (n=10). **I** The quantitative results of average fluorescence intensity (n=10). **J** The quantitative results of metastatic lung nodules (n=10). **K** Survival analysis between shnc-PLCG2 and sh-PLCG2 groups (n=10). Data were presented as mean±SD. ns no statistical significance, *P<0.05, **P<0.01, ***P<0.001, ****P<0.0001. oenc overexpression negative control, oe overexpression, shnc short hairpin RNA negative control, sh short hairpin RNA, HE hematoxylin-eosin staining.

To further demonstrate the biological functions of PLCG2 in promoting growth, invasion, and metastasis, as well as inhibiting apoptosis of CRC, we performed *in vivo* animal experiments. CRC cell lines transfected with the lentiviral vectors were injected subcutaneously into nude mice to construct nude mouse xenograft subcutaneous tumors. Macroscopic photographs of nude mice and subcutaneous tumors showed that tumors in PLCG2 overexpression group appeared to be significantly larger than those in the control group, whereas tumors in PLCG2 knockdown group showed reduced size (Figure 6A, B). The growth curves of subcutaneous tumors also showed that overexpression of PLCG2 promoted tumor growth *in vivo*, and knockdown of PLCG2 inhibited tumor growth (Figure 6C). The subcutaneous tumors of nude mice with overexpression of PLCG2 were significantly greater in volume and weight than those of the control group. In contrast, knockdown of PLCG2 greatly reduced the volume and weight of tumors (Figure 6D). The results of HE staining indicated that in comparison with the control group, some of the tumor cells of subcutaneous tumors in nude mice with knockdown of PLCG2 were more rounded, with deeply stained nuclei, condensed cytoplasm, light red cytoplasm, and clumped chromatin, which were apoptotic features. Meanwhile, some of them appeared condensed, with fragmented nuclei, nucleolus lysis, and coagulation or lysis of cytoplasm. These cells also exhibited deep red granularity staining, disintegration of the stroma, swelling, and breaking or liquefaction of collagen fibers, and fused with the necrotic cells to form a piece of granular, red-stained, unstructured material, which all represented signs of necrosis. IHC results further demonstrated that knockdown of PLCG2 significantly promoted the expression of E-cadherin, as well as apoptosis-promoting proteins (Bax and Cleaved Caspase-3), and inhibited the expression of the proliferation marker (Ki-67) and N-cadherin, whereas overexpression of PLCG2 showed the opposite effects (Figure 6E; Figure S6A, B). Then, we constructed the lung metastasis model by injecting tumor cells into the tail vein of nude mice. The results of *in vivo* fluorescence imaging and anatomical pictures of lungs in nude mice showed that knockdown of PLCG2 significantly inhibited the lung metastasis of CRC (Figure 6F). Pathological results of HE staining of mice lungs further confirmed the above results (Figure 6G). We conducted the chi-square test between the two groups and found that knockdown of PLCG2 significantly inhibited the incidence of distant lung metastasis in CRC cells (Figure 6H). Additionally, the intensity of *in vivo* fluorescence imaging (Figure 6I) and the number of lung metastatic nodules (Figure 6J) in nude mice with knockdown of PLCG2 were significantly decreased. 60-day survival analyses also indicated that nude mice with knockdown of PLCG2 survived significantly longer than those in the control group (Figure 6K).

### Functional enrichment analysis and experimental validation revealed that PLCG2 activated the downstream mTOR signaling pathway by phosphorylating Akt

**Figure 7:**
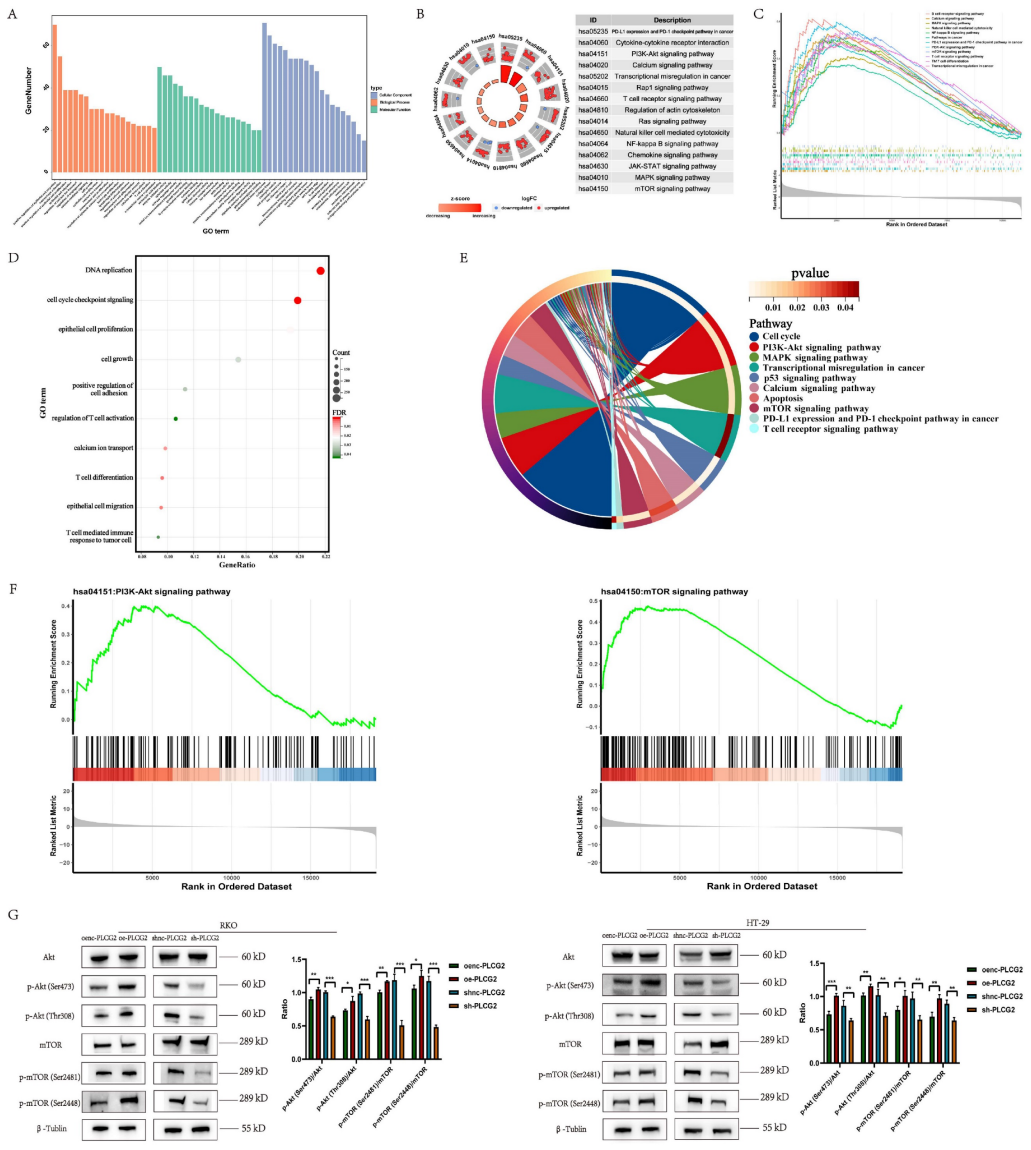
Functional enrichment analysis and experimental validation revealed that PLCG2 activated the downstream mTOR signaling pathway by phosphorylating Akt. **A** GO enrichment analysis of PLCG2-related differentially expressed genes in the TCGA-COREAD cohort. **B** KEGG enrichment analysis of PLCG2-related differentially expressed genes in the TCGA-COREAD cohort. **C** GSEA enrichment analysis of PLCG2-related differentially expressed genes in the TCGA-COREAD cohort. **D** In-house RNA-seq data from RKO cells transfected with lentivirus over-expressing PLCG2 were analyzed for GO enrichment (n=5). **E** In-house RNA-seq data from RKO cells transfected with lentivirus over-expressing PLCG2 were analyzed for KEGG enrichment (n=5). **F** In-house RNA-seq data from RKO cells transfected with lentivirus over-expressing PLCG2 were analyzed for GSEA enrichment (n=5). **G** The regulatory role of PLCG2 on the Akt-mTOR signaling pathway was verified by western blotting (n=3). Data were presented as mean±SD. ns no statistical significance, *P<0.05, **P<0.01, ***P<0.001, ****P<0.0001. oenc overexpression negative control, oe overexpression, shnc short hairpin RNA negative control, sh short hairpin RNA, PI3K phosphoinositide 3-kinase, Akt protein kinase B, mTOR mammalian target of rapamycin, p-Akt phosphorylated protein kinase B, p-mTOR phosphorylated mammalian target of rapamycin, GO Gene Ontology, KEGG Kyoto Encyclopedia of Genes and Genomes, GSEA Gene Set Enrichment Analysis.

We performed functional enrichment analysis to reveal the signaling pathways, biological processes, and molecular functions in which PLCG2 might be involved based on the TCGA dataset. GO-BP analysis indicated that "positive regulation of epithelial cell migration", "regulation of epithelial cell proliferation", "regulation of angiogenesis", "T cell activation" and "cell growth" were significantly enriched. GO-MF analysis showed that PLCG2 was associated with "receptor ligand activity", "signaling receptor activator activity" and "immune receptor activity". GO-CC analysis showed that PLCG2-related differentially expressed genes were located in "endoplasmic reticulum lumen", "membrane raft" and "secretory granule membrane" (Figure 7A). KEGG results showed that the "PI3K-Akt signaling pathway", "mTOR signaling pathway", "PD-L1 expression and PD-1 checkpoint pathway in cancer" and "T cell receptor signaling pathway" were significantly enriched (Figure 7B). Interestingly, further GSEA analysis showed that the "PI3K-Akt signaling pathway", "T cell receptor signaling pathway", "mTOR signaling pathway" and "PD-L1 expression and PD-1 checkpoint pathway in cancer" were significantly activated in PLCG2 high-expression group (Figure 7C). Subsequently, we performed RNA-seq in CRC cell lines overexpressing PLCG2 and similarly conducted functional enrichment analysis, with the results (Figure 7D and 7E) shown in the figures.

Interestingly, GSEA analysis showed that the "PI3K-Akt signaling pathway" and "mTOR signaling pathway" were significantly activated in PLCG2-overexpressing CRC cell lines (Figure 7F). Based on the results obtained from the functional enrichment analysis above, we took into account the vital roles that the "PI3K-Akt signaling pathway" and "mTOR signaling pathway" played in regulating tumor fate and signal transduction. Consequently, we verified the oncogenic activation effect of PLCG2 in the PI3K-Akt/mTOR signaling pathway. With the use of western blotting, we discovered that overexpression of PLCG2 could phosphorylate Akt at Ser473 and Thr308 sites, leading to the functional activation of Akt, and further activating the downstream mTOR signaling pathway by phosphorylating mTOR at its Ser2481 and Ser2448 sites, and the opposite effects were observed in knockdown of PLCG2 (Figure 7G).

### PLCG2 promoted CRC progression by activating the Akt-mTOR signaling pathway both *in vivo* and *in vitro*

**Figure 8:**
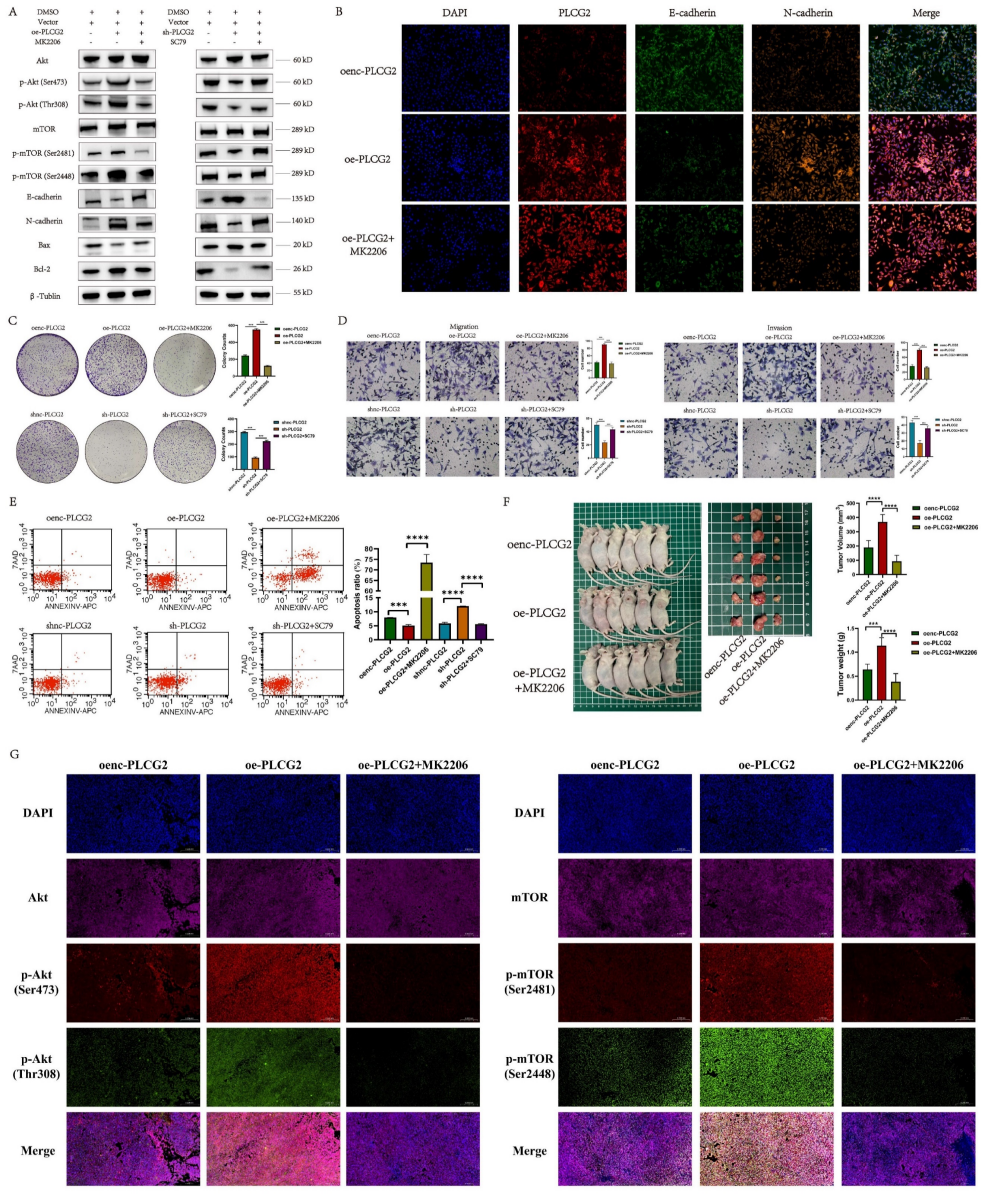
PLCG2 promoted CRC progression by activating Akt-mTOR signaling pathway both *in vivo* and *in vitro*. **A** The activity of Akt-mTOR signaling pathway and protein expression of E-cadherin, N-cadherin, Bax and Bcl-2 were detected by western blotting in PLCG2 over-expressing RKO cells treated with MK2206 and in PLCG2 knock-down RKO cells treated with SC79 (n=3). **B** The relationship between PLCG2 protein expression and the protein expression of E-cadherin and N-cadherin was verified by mIF assay (n=3). **C** The results of colony formation assay in PLCG2 over-expressing RKO cells treated with MK2206 (n=3). **D** The results of transwell assay in PLCG2 over-expressing RKO cells treated with MK2206 (n=3). **E** The apoptosis was detected by flow cytometry in PLCG2 over-expressing RKO cells treated with MK2206 (n=5). **F** Macroscopic observations of xenografted nude mice and subcutaneous tumors treated with MK2206 *in vivo* (n=6). **G** The volume and weight of subcutaneous tumors (n=6). **H** The activity of the Akt-mTOR signaling pathway was assessed by the mIF assay in subcutaneous tumor tissues (n=6). Data were presented as mean±SD. ns no statistical significance, *P<0.05, **P<0.01, ***P<0.001, ****P<0.0001. oenc overexpression negative control, oe overexpression, shnc short hairpin RNA negative control, sh short hairpin RNA, DMSO dimethyl sulfoxide, Akt protein kinase B, mTOR mammalian target of rapamycin, p-Akt phosphorylated protein kinase B, p-mTOR phosphorylated mammalian target of rapamycin.

As we recognized the importance of Akt-mTOR signaling in PLCG2 promoting CRC progression, we performed a series of rescue experiments *in vivo* and *in vitro*. Overexpression of PLCG2 activated Akt by phosphorylating it and then activated the downstream mTOR signaling pathway by phosphorylating mTOR, which led to the promotion of the expression of N-cadherin and apoptosis-suppressing protein (Bcl-2), and inhibition of the expression of E-cadherin and apoptosis-promoting protein (Bax), which both could be reversed by MK2206, an orally active Akt inhibitor. Knockdown of PLCG2 decreased Akt phosphorylation and suppressed Akt function, then decreased mTOR phosphorylation and suppressed the downstream mTOR signaling pathway, which inhibited the expression of N-cadherin and Bcl-2 and promoted the expression of E-cadherin and Bax, which could be reversed by SC79, an Akt activator (Figure 8A). The multicolor immunofluorescence (mIF) also confirmed that MK2206 could reverse the effect of PLCG2 overexpression on the expression of EMT markers (Figure 8B) and apoptosis-related proteins (Figure S7A). *In vitro* experiments demonstrated that MK2206 could reverse the biological effects of overexpression of PLCG2 on increasing tumor cell proliferation, migration, and invasion, and decreasing apoptosis, whereas SC79 could reverse the biological effects of knockdown of PLCG2 on regulating certain phenotypes (Figure 8C, D, E). Subcutaneous xenograft tumors in nude mice showed significantly greater tumor volume and weight in PLCG2 overexpression group (oe-PLCG2) than in the control group (oenc-PLCG2), but tumor volume and weight in oe-PLCG2 group that had undergone MK2206 administration (oe-PLCG2+MK2206) were significantly suppressed and lower than that in oe-PLCG2 group (Figure 8F). The mIHC results of subcutaneous tumor tissues indicated a significantly lower expression of phosphorylated Akt proteins (Ser473 and Thr308) and phosphorylated mTOR proteins (Ser2481 and Ser2448) in oe-PLCG2+MK2206 group in contrast to oe-PLCG2 group (Figure 8G). IHC showed that the expression of proliferation marker (Ki-67) and N-cadherin was significantly lower, while E-cadherin and apoptosis-promoting marker (Bax and Cleaved Caspase-3) had significantly higher expression in oe-PLCG2+MK2206 group compared to oe-PLCG2 group (Figure S7B, C). Overall, this result suggested that PLCG2 promoted tumor cell proliferation, invasion, metastasis, and EMT and inhibited apoptosis through activation of the Akt-mTOR signaling pathway, thereby facilitating CRC progression.

### High expression of PLCG2 induced the formation of tumor immunosuppressive microenvironment and facilitated tumor immune escape in CRC

To delve into the role of PLCG2 in the TME, we explored the relationship between PLCG2 expression and the infiltration of various immune cells and immune-related scores in the TCGA-COREAD cohort based on various bioinformatics algorithms. In terms of immune-related scores, CRC patients in the high PLCG2 expression group were found to have lower cytolytic score, inflammation score, StromalScore, InmuneScore, and ESTIMATEScore compared to those in the other group (Figure S8A). Subsequently, algorithms such as quanTIseq, TIMER, EPIC, and CIBERSORT were used, which demonstrated that immune cell infiltration between two different PLCG2 expression groups differed significantly. In the high PLCG2 expression group, there was significantly more infiltration of immunosuppressive cells such as M2 macrophages and Tregs and significantly less infiltration of NK cells and CD8^+^ T cells (Figure S8B). Then, we investigated the correlation between PLCG2 expression and various classical immune cells based on the results of the above bioinformatics algorithms, which showed that PLCG2 expression was upregulated with greater Treg cell infiltration and less CD8^+^ T cell infiltration (Figure S8C). As the high expression of immune checkpoints (ICs) on the surface of tumor cells and immune cells contributed significantly to tumor immune escape, we investigated the expression of ICs in CRC patients with different PLCG2 expressions. Overall, CRC patients with high PLCG2 expression had higher expression of ICs such as CD274, CTLA4, IDO1, and PDCD1 expression (Figure S8D).

**Figure 9:**
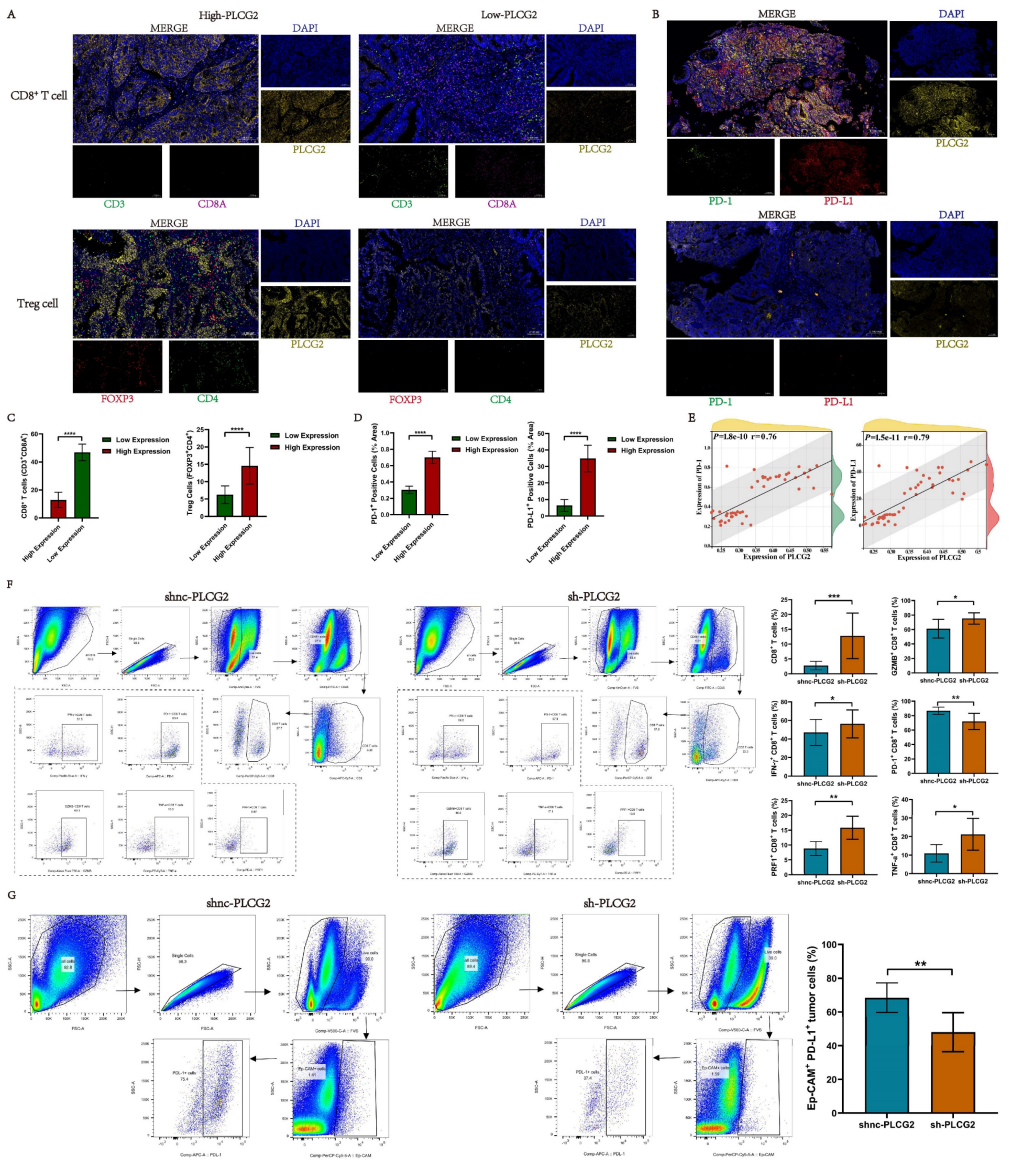
High expression of PLCG2 induced infiltration of Treg cells, promoted expression of immune checkpoints PD-1 and PD-L1, and suppressed infiltration and functional activation of CD8+ T cells. **A** Validation of the relationship between PLCG2 and Treg cells as well as CD8+ T cells infiltration by mIHC experiments in CRC clinical samples (n=40). **B** Validation of the relationship between PLCG2 expression and the expression of PD-1 and PD-L1 in CRC clinical samples by mIHC experiments (n=40). **C** The quantitative results of Treg cells and CD8+ T cells infiltration (n=40). **D** The quantitative results of PD-1+ positive cells and PD-L1+ positive cells (n=40). **E** Correlation of PLCG2 expression with PD-1 and PD-L1 expression was verified based on the results of mIHC experiments (n=40).** F** Infiltration of CD8+ T cell subsets in subcutaneous tumor tissues of C57BL/6 mice was detected by flow cytometry (n=8). **G** The expression of PD-L1 on the surface of tumor cells in subcutaneous tumor tissues of C57BL/6 mice was detected by flow cytometry (n=8). Data were presented as mean±SD. ns no statistical significance, *P<0.05, **P<0.01, ***P<0.001, ****P<0.0001. oenc overexpression negative control, oe overexpression, shnc short hairpin RNA negative control, sh short hairpin RNA, GZMB granzyme B, IFN-γ interferon-gamma, PRF1 perforin 1, TNF-α tumor necrosis factor α.

Based upon the results of the above bioinformatics analysis, preliminary studies revealed that high PLCG2 expression was strongly tied to the tumor immunosuppressive microenvironment in CRC, and high PLCG2 expression might induce the infiltration of Treg cells and restrained CD8^+^ T cell infiltration. Meanwhile, high expression of PLCG2 might promote the expression of PD-L1 and PD-1, thus hampering the killing effect of T lymphocytes on tumor cells, diminishing the efficacy of immunotherapy, and facilitating immune escape. Therefore, we collected pathological tissues from CRC patients at our medical center to conduct mIHC experiments to further verify whether PLCG2 expression could impact the infiltration of CD8^+^ T cells and Treg cells, as well as PD-L1 and PD-1 expression. The results showed that CRC patients with high expression of PLCG2 had more infiltration of Treg cells (FOXP3^+^CD4^+^) and less infiltration of CD8^+^ T cells (CD3^+^CD8A^+^) in the TME compared to those with low expression of PLCG2 (Figure 9A, C). Additionally, PLCG2 could significantly promote the expression of PD-L1 and PD-1 (Figure 9B, D), and there was a positive correlation (Figure 9E). Subsequently, the subcutaneous xenograft tumor model was constructed in C57BL/6 mice with normal immune system, and the immune cell infiltration in the TME and the expression of immune checkpoints were investigated with flow cytometry. The results showed that the total CD8^+^ T cell infiltration in the tumor immune microenvironment was significantly increased in the knockdown of PLCG2 group (sh-PLCG2), with the significant increase of GZMB^+^CD8^+^ T cells, PRF1^+^CD8^+^ T cells, TNF-α^+^CD8^+^ T cells and IFN-γ^+^CD8^+^ T cells, and the significant decrease of PD-1^+^CD8^+^ T cells, compared to the control group (shnc-PLCG2) (Figure 9F). There were significantly fewer PD-L1^+^Ep-CAM^+^ tumor cells in the knockdown of PLCG2 group (sh-PLCG2) than in the control group (shnc-PLCG2) (Figure 9G). It was evident that PLCG2 could promote the formation of the immunosuppressive microenvironment by inducing Treg cell infiltration and restraining CD8^+^ T cell infiltration. Furthermore, PLCG2 could promote the expression of PD-L1 and PD-1, and suppress the functional activation of CD8^+^ T cells to enhance the tumor immune escape. In summary, PLCG2 might be an important biomarker and therapeutic target for orchestrating the immunosuppressive microenvironment and suppressing tumor immune escape.

### Prediction of immunotherapy response in CRC patients based on PLCG2 expression and screening of small molecule compounds targeting PLCG2

PLCG2 was closely associated with tumor immunosuppressive microenvironment, but whether PLCG2 could influence the response of CRC patients to immunotherapy remained unclear. Firstly, the high PLCG2 expression group had a lower immunophenotype score (IPS) compared to the low PLCG2 expression group (Figure S9A). There was a higher percentage of MSI-H patients in the low PLCG2 expression group compared to the high PLCG2 expression group (Figure S9B), and CRC patients with dMMR tended to have lower expression of PLCG2 compared to patients with pMMR (Figure S9C). CRC patients in the low PLCG2 expression group also had higher TMB compared to the high PLCG2 expression group (Figure S9D). In the TCGA-COREAD cohort, a positive correlation was found between PLCG2 expression and the expression of CD274, CTLA4, PDCD1, and LAG3 (Figure S9E). Further, in the Ruijin cohort, patients with dMMR had lower expression of PLCG2 than patients with pMMR (Figure S9F).

To investigate whether PLCG2 was associated with chemotherapy sensitivity in CRC patients, we predicted the IC50 of drugs in two different PLCG2 expression groups and discovered that CRC patients with low PLCG2 expression might be more sensitive to gemcitabine, and those with high PLCG2 expression might be more sensitive to cisplatin, docetaxel, etoposide and methotrexate (Figure S9G). Subsequently, we screened and identified small molecule compounds targeting PLCG2 protein by the CMap drug database, and the molecular mechanism of action of these small molecule compounds involved ACE inhibitors, benzodiazepine receptor agonists, calcium channel blockers, and NF-κB pathway inhibitors (Figure S9H). Figure S9I illustrated the 3D chemical structures of the top 6 small molecule compounds with the highest predicted scores. The top-ranked small molecule compound D-4476, a known highly selective, cell-penetrating casein kinase 1 (CK1) and TGF-β1 receptor (TGFBR1) inhibitor, was selected for molecular docking and visualization with PLCG2 proteins (Figure S9J).

### Knock-down of PLCG2 potentiated the efficacy of ICB therapy for CRC and synergistic therapy suppressed tumors for improved prognosis

Given the oncogenic effects of PLCG2 on tumor cells and its role as an important contributor to the development of the tumor immunosuppressive microenvironment and the enhancement of immune escape, we hypothesized that targeted inhibition of PLCG2 expression could not only impede the malignant phenotype of tumor cells but also enhance the efficacy of anti-PD-1 therapy.

**Figure 10:**
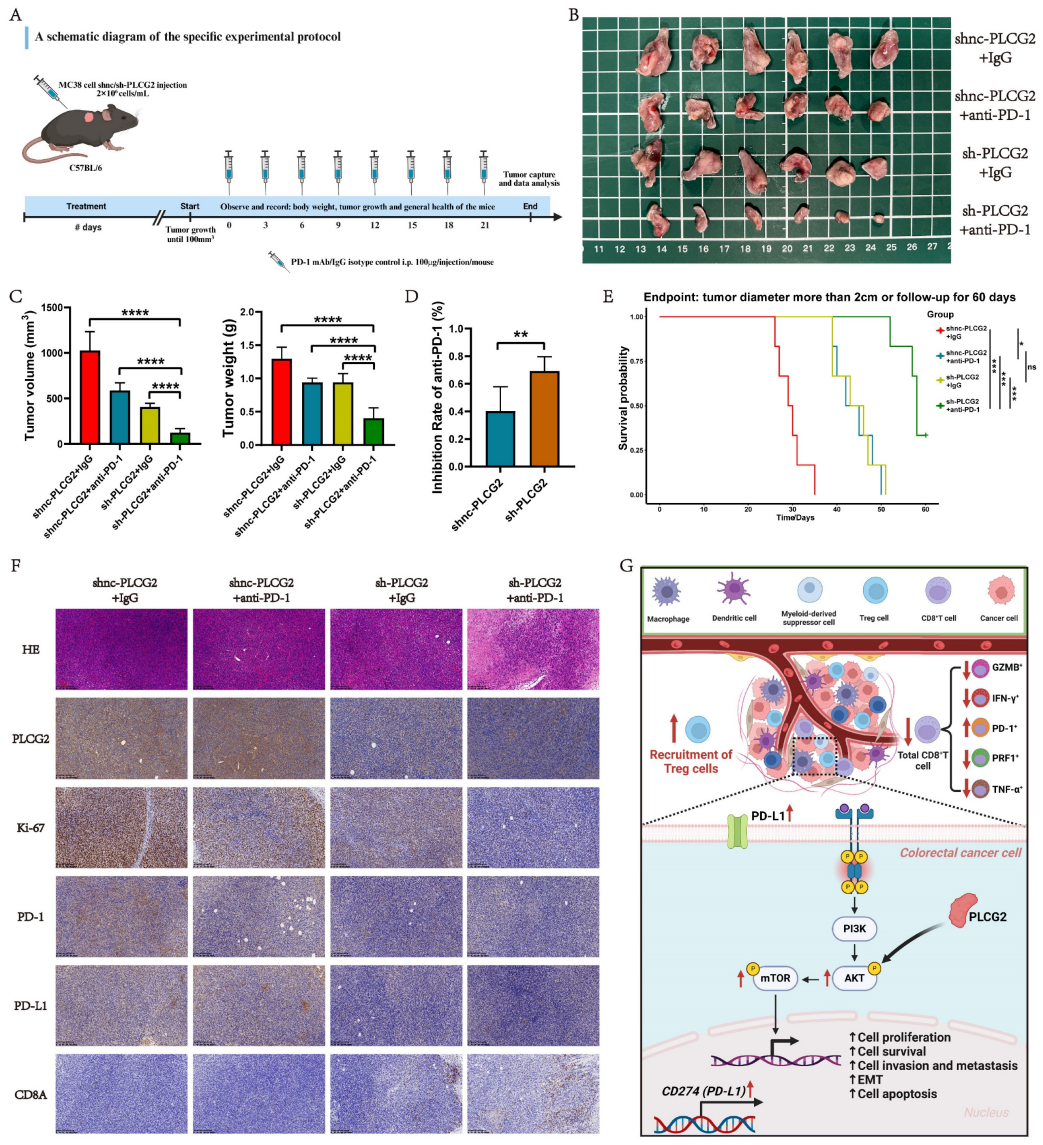
Knockdown of PLCG2 potentiated the efficacy of ICB therapy for CRC and synergistic therapy suppressed tumors for improved prognosis. **A** A schematic diagram of the specific experimental protocol. **B** Macroscopic observation of subcutaneous tumor tissue at the endpoint of the experiment (n=6). **C** The volume and weight of subcutaneous tumors (n=6).** D** Tumor inhibition rate in each group after anti-PD-1 therapy (n=6). **E** Survival analysis of C57BL/6 mice in each group (n=6). **F** Representative IHC images of subcutaneous tumor tissue (n=6). **G** Summary of the study and schematic diagram of the mechanism. Data were presented as mean±SD. ns no statistical significance, *P<0.05, **P<0.01, ***P<0.001, ****P<0.0001. shnc short hairpin RNA negative control, sh short hairpin RNA, EMT epithelial mesenchymal transition, i.p. intraperitoneal injection, HE hematoxylin-eosin staining, GZMB granzyme B, IFN-γ interferon-gamma, PRF1 perforin 1, TNF-α tumor necrosis factor α.

Hence, synergistic therapy could significantly remodel the tumor immunosuppressive microenvironment and suppress the functional exhaustion of CD8^+^ T cells, thus inhibiting the tumor immune escape. To confirm this hypothesis, we constructed the subcutaneous xenograft tumor model in C57BL/6 mice and randomly grouped them according to body weight and tumor volume for administration of the drug (Figure 10A). Figure 10B presented the subcutaneous tumors in each group at the endpoint of the experiment. *In vivo* experiments showed that the tumor volume and weight of the synergistic therapy group (sh-PLCG2+anti-PD-1) were significantly lower than those of other groups (Figure 10C). To determine the potentiating effect of knockdown of PLCG2 on anti-PD-1 therapy, we compared the tumor inhibition rate of this therapy in the control group (shnc-PLCG2) with that in the knockdown group (sh-PLCG2). The results indicated that the synergistic therapy group (sh-PLCG2+anti-PD-1) exhibited higher tumor inhibition rate (Figure 10D). Subsequently, we conducted follow-up and survival analyses of the mice, which suggested that the synergistic therapy group (sh-PLCG2+anti-PD-1) had a better prognosis (Figure 10E). The IHC results of subcutaneous tumors demonstrated that synergistic therapy significantly decreased the expression of the tumor proliferation marker (Ki-67) and the immune checkpoints (PD-1 and PD-L1) but significantly promoted the expression of CD8A, suggesting that synergistic therapy could effectively inhibit tumor proliferation, induce the infiltration of CD8^+^ T cells and reverse the tumor immune escape (Figure 10F; Figure S10A, B).

## Discussion

Colorectal cancer (CRC) is a worldwide prevalent malignancy with high morbidity and mortality. Investigating the underlying molecular mechanisms of the tumorigenesis and progression of CRC is crucial for improving its diagnosis and therapeutic strategies. In this study, we revealed PLCG2 as a prognostic factor for CRC with a prospective clinical observational study, uncovered the role of PLCG2 in shaping the heterogeneity of CRC microenvironment and examined in detail the biological function of PLCG2 and its molecular mechanism. Additionally, we clarified the immunomodulatory role of PLCG2 in inducing tumor immunosuppressive microenvironment and thus enhancing tumor immune escape and explored the efficacy of the synergistic therapy with knockdown of PLCG2 and ICB therapy, which identified PLCG2 as a potentially effective biomarker and a valuable target for the early diagnosis and individualized therapy of CRC (Figure 10G).

Previous studies have shown that PLCG2 was highly expressed in various malignant tumors. Chan *et al.* identified that the phenotype in small-cell lung cancer with high expression of PLCG2 characterized by stem cell-like metastatic features was associated with poor overall survival 19. Li *et al.* found that PLCG2, which was associated with the EGFR pathway, was a risk factor for gastric cancer and esophageal squamous cell carcinoma in the Chinese population and was significantly highly expressed in those cancers 20. Fei *et al.* suggested that the expression of phagocytosis-associated gene PLCG2 was increased in low-grade glioma and strongly associated with worse survival 21. Qi *et al.* observed that PLCG2 was significantly upregulated in the cohort of triple-negative breast cancer patients and had important interactions with the molecular mechanism of triple-negative breast cancer-associated BRCA1 22. Zhang *et al.* discovered a remarkable increase in PLCG2 expression in colon cancer and suggested that this gene could be a prognostic risk factor 23. Thus, PLCG2 has a unique expression profile in malignant tumors. In this study, pan-cancer analysis showed that PLCG2 was highly expressed in a variety of cancers, especially in CRC cell lines, tissues, and organoids. Moreover, its expression level correlated positively with poor prognosis and advanced clinicopathological features (pT, pM, pTNM stage, and tumor site) in both public databases and the prospective clinical cohort at our center, suggesting that it might play an oncogenic role.

PLCG2 has an important role in malignant tumors, including hematological and solid tumors, and could affect tumorigenesis and progression through a variety of molecular mechanisms. PLCG2 was strongly associated with the development of leukemia, and its activity was a potential biomarker of response in CLL and diffuse large B-cell lymphoma (DLBCL) treatment. PLCG2 and/or Btk mutations have been reported to be present in approximately 11% to 90% of ibrutinib-refractory CLL cases, and detection of PLCG2 mutations could be valuable in predicting early relapse in ibrutinib-treated CLL patients 24. Xiao *et al.* found that chromophobe renal cell carcinoma (chRCC) cells relied on extracellular macromolecules as a source of amino acids to maintain cell proliferation and survival through activation of endocytosis. Such activation for amino acid uptake in chRCC cells was greatly impaired when the PLCG2/IP3/Ca^2+^/PKC was inhibited 25. In non-small cell lung cancer (NSCLC), PLCG2 was correlated with HMGB1 expression and was a biomarker for predicting patient survival and progression 26. Targeted inhibition of PLCG2 attenuated glioma growth, invasion, and metastasis 27. There was also a strong association between PLCG2 and tumor drug resistance. In BRCA wild-type high-grade serous ovarian cancer, PLCG2 might be a potent biomarker for predicting the response of patients to first-line chemotherapy 28. Stanislaus *et al.* found that knockdown of PLCG2 sensitized human cervical adenocarcinoma cells to doxorubicin and paclitaxel 29. Currently, only a few relevant studies have focused on the role of PLCG2 in CRC. Hence, the biological function of PLCG2 in CRC still needs to be further revealed. In this study, we conducted a series of experiments to clarify that PLCG2, as an oncogene of CRC, significantly promoted CRC proliferation, invasion, metastasis, and EMT, regulated the cell cycle, and inhibited apoptosis. Subsequently, we used transcriptome sequencing and various molecular biology techniques to elucidate the mechanism by which PLCG2 accelerated the malignant progression of CRC through activation of the Akt-mTOR pathway both *in vivo* and *in vitro*. The Akt-mTOR signaling pathway played a central role in cellular physiology and pathology, particularly in the tumorigenesis and progression of CRC, and its aberrant activation had profound effects on several key biological properties of tumors. It was worth noting that activation of this signaling pathway promoted the proliferation of tumor cells and increased the frequency of cell division by accelerating cell cycle progression 30. At the same time, the Akt-mTOR signaling pathway enhanced the anti-apoptotic ability of tumor cells by inhibiting pro-apoptotic signaling, enabling them to survive in unfavorable conditions such as hypoxia and nutrient deficiency 31. With respect to tumor invasion and metastasis, the pathway potentiated the invasiveness of tumor cells, enabling them to penetrate the basement membrane and enter the blood circulation to form distant metastases 32. The signaling pathway also accelerated tumor angiogenesis by promoting the proliferation and migration of vascular endothelial cells, which provided the tumor with essential oxygen and nutrients, further promoting tumor growth and proliferation 33.

In the 19th century, Stephen Paget described the close connection between the TME and tumor cells with the "seed and soil" theory, indicating the important role of TME in tumor development 34. The TME was characterized by hypoxia, low pH, interstitial hypertension, and hyperpermeability of the tumor vasculature 35. Tumor cells could influence the TME by secreting cellular signaling molecules, initiating tumor angiogenesis, and mediating immune tolerance, while immune cells could influence tumor cell proliferation, invasion, and metastasis 36. The TME of CRC was a highly heterogeneous ecosystem composed of multiple cell types, extracellular matrix, immune cells, vascular system, and secreted bioactive molecules. These components collectively regulated the proliferation, survival, invasion, and metastasis of tumor cells through complex interactions such as direct cell-to-cell contact, exchange of secreted factors, immune regulation, angiogenesis, metabolic reprogramming, activation of signaling pathways, development of drug resistance, inflammatory response, and genetic instability 37. Fibroblasts, immune cells, and endothelial cells in the tumor microenvironment promoted tumor angiogenesis and immune escape, while tumor cells adapted to hypoxic and nutrient-deficient environments through metabolic reprogramming. In addition, the TME facilitated tumor heterogeneity and evolution by influencing DNA repair mechanisms and increasing the genetic instability of tumor cells 38. These mechanisms not only influenced the biological behaviors of CRC but also provided novel targets for therapy. However, the part played by PLCG2 in the CRC tumor microenvironment and the regulatory mechanisms involved remained elusive. In this study, we found a positive correlation that the expression of PLCG2 was significantly increased with the upregulation of immune checkpoints such as CD274, PDCD1, LAG3 and CTLA4 via bioinformatics analysis and observed that PLCG2 could promote the formation of tumor immunosuppressive microenvironment by inducing the infiltration of immune-suppressing cells (M2 macrophages and Treg cells) and inhibiting the infiltration of immune-activating cells (NK cells and CD8^+^ T cells). Using the CRC tissue microarrays from our center, we subsequently verified how PLCG2 expression was tied to immune cell infiltration as well as PD-1 and PD-L1 expression, and the mIHC results demonstrated that high expression of PLCG2 led to low CD8^+^ T-cell infiltration and high Treg-cell infiltration, as well as abundant expression of PD-1 and PD-L1. To pinpoint how PLCG2 induced the formation of the CRC immunosuppressive microenvironment and facilitated tumor immune escape, we constructed subcutaneous tumor models with the MC38 cell line and collected tumor tissues for flow cytometry, which demonstrated that knockdown of PLCG2 significantly promoted total CD8^+^ T -cell infiltration, and also induced the infiltration of GZMB^+^CD8^+^ T cells, PRF1^+^CD8^+^ T cells, IFN-γ^+^CD8^+^ T cells and TNF-α^+^CD8^+^ T cells, and inhibited PD-1^+^CD8^+^ T cell infiltration. Knockdown of PLCG2 significantly inhibited the tumor cell surface expression of PD-L1. This indicated that PLCG2 might be an essential factor in predicting and remodeling the CRC immune microenvironment, especially in close relation to CD8^+^ T cells, and might be an important molecular target for modulating CD8^+^ T cells as well as for reversing CRC immune escape.

Immunotherapy has become the fifth most important tumor treatment after surgery, chemotherapy, radiotherapy and molecular targeted therapy 39, revolutionizing the therapeutic approach in CRC. The KEYNOTE-177 trial was the most important first-line treatment clinical trial to date, confirming that pembrolizumab treatment could be the first-line standard treatment for dMMR and MSI-H CRC 40-42. Although immunotherapy has manifested greater curative power with fewer side effects than chemotherapy in many trials and clinical studies, and the objective remission rate (ORR) of MSI-H/dMMR CRC patients receiving immunotherapy has been maintained at 30-70%, the applicability and efficacy of MSI-H/dMMR as the currently recognized biomarker of immunotherapy in CRC was not satisfactory 43. On the one hand, the proportion of MSI-H/dMMR in CRC patients was only approximately 20%, and less than 5% in patients with advanced CRC. On the other hand, some MSI-H/dMMR patients remained non-responsive and were often resistant to immunotherapy, while some non-MSI-H/dMMR patients could still benefit from immunotherapy 44. To avoid the possibility of inappropriate or over-treatment, we recognized that urgent efforts are required to overcome the dilemma in which the current immunotherapy suffers from a lack of reliable biomarkers to predict its effectiveness in CRC patients and potentiate its efficacy. Given the important function of PLCG2 in remodeling CRC tumor microenvironment, we explored the relationship between PLCG2 and immunotherapy response in CRC. First, we carried out bioinformatics analysis to find that in the TCGA-COREAD cohort, patients with low expression of PLCG2 had higher IPS scores, as well as higher TMB. Compared with pMMR patients, dMMR patients tended to have low expression of PLCG2. In the Ruijin cohort at our centre, we similarly found that dMMR patients had lower expression of PLCG2. This implied that CRC patients with low expression of PLCG2 might benefit more from immunotherapy. Subsequently, we found that knockdown of PLCG2 significantly potentiated the efficacy of anti-PD-1 therapy, which suppressed the growth of subcutaneous tumors and conferred better survival rate in the mice with subcutaneous tumors. Furthermore, the immunohistochemical results suggested that the synergistic therapy with PLCG2 knockdown and anti-PD-1 could reverse tumor immune escape by inhibiting the expression of immune checkpoints and induce CD8^+^ T cells infiltration to transform the tumor microenvironment from "cold" to "hot". Our study demonstrated that PLCG2 might be a promising biomarker and target for predicting the effectiveness of immunotherapy in CRC patients as well as for potentiating the efficacy of anti-PD-1 therapy.

In summary, our study reported a significant increase in PLCG2 expression in CRC, which was closely linked to poor prognosis and advanced clinicopathological features of patients. Acting as an oncogene of CRC, PLCG2 significantly promoted CRC proliferation, invasion, metastasis, and EMT and inhibited apoptosis. PLCG2 was also found to be an essential regulator of CRC tumor microenvironment as it could inhibit immune-activated cell infiltration (CD8^+^ T cells) and promote immune-suppressed cell infiltration (Treg cells) and the expression of immune checkpoints, leading to the immune escape of CRC. Furthermore, knockdown of PLCG2 potentiated the efficacy of anti-PD-1 therapy for CRC, and targeted inhibition of PLCG2 in combination with ICB therapy might confer better immunotherapy response and afford CRC patients the potential for survival advantages. This is the first study to identify that PLCG2 could be considered a precise biomarker and promising target for predicting CRC prognosis, individualized treatment, reversing tumor immune escape and overcoming the resistance to ICB therapy. However, despite these prospective findings, the translation of PLCG2-targeted therapy into clinical practice still posed considerable challenges, which were also unavoidable limitations of this study. First, we need to expand the sample size of the clinical cohort by enrolling more CRC patients and further evaluate the clinical value of PLCG2 as an effective biomarker and therapeutic target for CRC in future multicenter prospective clinical studies. To overcome this limitation, our research team has been collaborating with an increasing number of medical centers and enrolling more patients based on the previous clinical trials. Secondly, the molecular mechanisms of PLCG2 in CRC and TME, especially the modulation of immune checkpoints and CD8^+^ T cells, require in-depth explorations to clarify the crucial role of PLCG2 and to discover more therapeutic targets, which is also the focus of our subsequent research. Finally, as there are currently no specific inhibitors targeting PLCG2 available, our team is now attempting to screen for compounds that can target PLCG2 protein and thereby inhibit PLCG2 protein expression, activity, or function. Recognizing the potential translational values of PLCG2 inhibitors, we will further evaluate the efficacy and clinical feasibility of PLCG2 inhibitors alone or in combination with ICB therapy in preclinical animal models of CRC.

## Conclusions

In summary, we have identified for the first time that PLCG2 served as a precise biomarker and potential therapeutic target of CRC and revealed the biological function and mechanism of PLCG2 in promoting the malignant progression of CRC. Moreover, we clarified the role of PLCG2 in remodeling the immunosuppressive microenvironment and explored the translational value of targeting PLCG2 to potentiate the efficacy of ICB therapy and improve the prognosis for CRC in preclinical animal models.

## Supplementary Material

Supplementary figures and table.

## Figures and Tables

**Table 1:** The association between PLCG2 expression and the clinicopathological characteristics in the Ruijin cohort

Characteristics	Group		χ^2^	*P* value
	Low	High		
Gender				
Male	24	25	0.005	0.945
Female	13	14		
Age (years-old)				
<60	13	16	0.502	0.479
≥60	25	22		
Primary tumor site				
Left-hemi	23	18	4.648	0.031^*^
Right-hemi	11	24		
Tumor size (cm)				
<5	28	10	7.773	0.005^*^
≥5	16	22		
Pathological differentiation degree				
High to moderate	30	21	8.160	0.004^*^
Low	6	19		
pT				
pT1-3	30	19	6.951	0.008^*^
pT4	8	19		
pN				
pN0	27	23	0.422	0.516
pN1-2	12	14		
pM				
pM0	56	16	8.151	0.004^*^
pM1	0	4		
Vessel Carcinoma Embolus				
No	25	30	2.737	0.098
Yes	14	7		
Nerve invasion				
No	33	25	0.857	0.354
Yes	8	10		
EGFR				
Negative	16	9	13.187	<0.001^*^
Positive	11	40		
Ki-67				
<80%	31	17	5.932	0.015^*^
≥80%	10	18		
Microsatellite status				
MSS	11	28	7.549	0.006^*^
MSI	22	15		

*This symbol indicates a statistically significant difference (*P* <0.050).

## Abbreviations

PLCG2: phospholipase Cγ2
ICB: immune checkpoint blockade
CRC: colorectal cancer
IP3: inositol triphosphate
DAG: diacylglycerol
CLL: chronic lymphocytic leukemia
EMT: epithelial-mesenchymal transition
t-SNE: t-Distributed Stochastic Neighbor Embedding
IF: immunofluorescence
IHC: immunohistochemistry
AOD: average optical density
SPF: specific pathogen-free
mIHC: multiplex immunohistochemistry
mIF: multicolor immunofluorescence
IC: immune checkpoint
IPS: immunophenotype score
DLBCL: diffuse large B-cell lymphoma
chRCC: chromophobe renal cell carcinoma
NSCLC: non-small cell lung cancer
TME: tumor microenvironment
