# Supplementary material for: Targeting PLCG2 Suppresses Tumor Progression, Orchestrates the Tumor Immune Microenvironment and Potentiates Immune Checkpoint Blockade Therapy for Colorectal Cancer
Source: Int J Biol Sci. 2024 Oct 14;20(14):5548–75. doi: 10.7150/ijbs.98200 (PMC11528457; doi:10.7150/ijbs.98200)
Supplement: Supplementary file 1 — Supplementary figures and table. [file ijbsv20p5548s1.pdf]

Figure S1 Mutational landscape analysis in CRC between the high and low PLCG2 expression groups. **A** The frequency and type of PLCG2 mutations in pan-cancers. **B** The association of four CRC molecular subtypes with PLCG2 mutations. **C** Survival analysis of PLCG2 mutated and unmutated CRC patients. **D** The mutations on different protein structural domains of PLCG2. **E** Mutational landscapes of PLCG2 high-expression groups as well as mutational co-occurrence and mutually exclusive. **F** Mutational landscapes of PLCG2 low-expression groups as well as mutational co-occurrence and mutually exclusive. **G** Signaling pathways enriched by mutated genes in PLCG2 high-expression group. **H** Signaling pathways enriched by mutated genes in PLCG2 low-expression group. Data were presented as mean±SD. ns no statistical significance, $^{*}P<0.05$, $^{**}P<0.01$, $^{***}P<0.001$, $^{****}P<0.0001$.

Figure S2 PLCG2 expression was correlated with prognosis of CRC patients. **A** Survival analysis and survival status plot in the TCGA-COREAD cohort (n=499). **B** Survival analysis and survival status plot in the GSE39582 cohort (n=510). **C** Representative IHC images of high and low PLCG2 expression in the Ruijin cohort (n=68). **D** Survival analysis and survival status plot in Ruijin cohort. **E** The ROC curves of PLCG2 expression predicting prognosis in CRC patients. Data were presented as mean±SD. ns no statistical significance, $^{*}P<0.05$, $^{**}P<0.01$, $^{***}P<0.001$, $^{****}P<0.0001$. ROC receiver operating characteristic curve, AUC area under the curve

Figure S3 PLCG2 expression was correlated with clinicopathological features of CRC patients. **A** The complex heatmap was employed to show the distribution of PLCG2 expression and clinicopathological features (Status, Age, Gender, pT, pN, pM, Stage, Location) in TCGA-COREAD cohort. **B** The association between PLCG2 expression and pT, pM, Stage, Location in TCGA-COREAD cohort. **C** The association between PLCG2 expression and pT, pM, Stage, Location in GSE39582 cohort. **D** The association between PLCG2 expression and pT, pM, Stage, Location in Ruijin cohort. Data were presented as mean±SD. ns no statistical significance, $^{*}P<0.05$, $^{**}P<0.01$, $^{***}P<0.001$, $^{****}P<0.0001$. AOD average optical density

Figure S4 Single-cell profiles of colorectal cancer. **A** The single-cell clustering according to patient ID number (n=6). **B** 33,538 cells were clustered into 23 cell subpopulations based on the t-SNE dimensionality reduction algorithm. **C** The

annotation of cell subpopulations with the "SingleR" R package. **D** The expression of PLCG2 in different cell subpopulations. Data were presented as mean±SD. ns no statistical significance, $^{*}P<0.05$, $^{**}P<0.01$, $^{***}P<0.001$, $^{****}P<0.0001$.t-SNE t-Distributed Stochastic Neighbor Embedding, PLCG2 phospholipase Cγ2

Figure S5 Spatial transcriptome described the spatial distribution of PLCG2. **A** The HE staining of CRC tissue specimen. **B** The spots of the spatial transcriptome were divided into seven regional subgroups and the regional subgroups were annotated according to the classical cell markers. **C** The regional subgroups based on t-SNE downscaling and clustering. **D** The spatial distribution of PLCG2. **E** The PLCG2 expression in regional subgroups based on t-SNE downscaling and clustering. **F** The PLCG2 expression in the spatial regional subgroups that was annotated. **G** The violin plot displaying PLCG2 expression in different spatial regional subgroups. Data were presented as mean±SD. ns no statistical significance, $^{*}P<0.05$, $^{**}P<0.01$, $^{***}P<0.001$, $^{****}P<0.0001$. PLCG2 phospholipase Cγ2, HE hematoxylin-eosin staining

Figure S6 IHC experiments on subcutaneous tumors of xenografts in nude mice. **A** Representative IHC images of oenc-PLCG2 group (n=5) and oe-PLCG2 group (n=5). **B** The quantitative analysis of IHC experiments in oenc-PLCG2 group (n=5) and oe-PLCG2 group (n=5). Data were presented as mean±SD. ns no statistical significance, $^{*}P<0.05$, $^{**}P<0.01$, $^{***}P<0.001$, $^{****}P<0.0001$. oenc overexpression negative control, oe overexpression, HE hematoxylin-eosin staining

Figure S7 Multicolor immunofluorescence (mIF) and immunohistochemistry for rescue experiments with MK2206 treatment. **A** Cell mIF experiments in oenc-PLCG2 group (n=3), oe-PLCG2 group (n=3) and oe-PLCG2+MK2206 group (n=3). **B** Representative IHC images of oenc-PLCG2 group (n=6), oe-PLCG2 group (n=6) and oe-PLCG2+MK2206 group (n=6). **C** The quantitative analysis of IHC experiments in oenc-PLCG2 group (n=6), oe-PLCG2 group (n=6) and oe-PLCG2+MK2206 group (n=6). Data were presented as mean±SD. ns no statistical significance, $^{*}P<0.05$, $^{**}P<0.01$, $^{***}P<0.001$, $^{****}P<0.0001$. oenc overexpression negative control, oe overexpression

Figure S8 High expression of PLCG2 induced the formation of tumor

immunosuppressive microenvironment and facilitated tumor immune escape in CRC based on bioinformatics analysis. **A** Cytolytic score, Inflammation score, Immune and Stromal Score in high and low PLCG2 expression groups of TCGA-COREAD cohort. **B** Analysis of immune cell infiltration based on quanTIseq, TIMER, EPIC and CIBERSORT algorithms. **C** Correlation between PLCG2 expression and immune cell infiltration. **D** The expression of ICs in high and low PLCG2 expression groups of TCGA-COREAD cohort. Data were presented as mean±SD. ns no statistical significance, $^{*}P<0.05$, $^{**}P<0.01$, $^{***}P<0.001$, $^{****}P<0.0001$.

Figure S9 Prediction of immunotherapy response in CRC patients based on PLCG2 expression and screening of small molecule compounds targeting PLCG2. **A** IPS Score in high and low PLCG2 expression groups of TCGA-COREAD cohort. **B** The proportions of MSS, MSI-L and MSI-H status in high and low PLCG2 expression groups of TCGA-COREAD cohort. **C** The differences in PLCG2 expression of CRC patients with pMMR and dMMR status in TCGA-COREAD cohort. **D** The TMB in high and low PLCG2 expression groups of TCGA-COREAD cohort. **E** Correlation between PLCG2 expression and the expression of CD274, CTLA4, PDCD1 and LAG3. **F** Representative IHC images and the differences in PLCG2 expression of CRC patients with pMMR and dMMR status (n=76). **G** The differences in sensitivity to common chemotherapeutic drugs between CRC patients with high expression of PLCG2 and those with low expression of PLCG2. **H** The molecular mechanism of action of these small molecule compounds targeting PLCG2 protein by the CMap drug database. **I** The 3D chemical structures of the top 6 small molecule compounds with the highest predicted scores. **J** Molecular docking of the top-ranked small molecule compound D-4476 with PLCG2 protein. Data were presented as mean±SD. ns no statistical significance, $^{*}P<0.05$, $^{**}P<0.01$, $^{***}P<0.001$, $^{****}P<0.0001$. AOD average optical density, IC50 half maximal inhibitory concentration

Figure S10 The quantitative analysis of IHC experiments for synergistic therapy with PLCG2 knockdown and anti-PD-1. **A** The quantitative analysis of IHC experiments for PLCG2 protein (n=6) and Ki-67 protein (n=6). **B** The area ratio of PD-1^{+} cells (n=6), PD-$L1^{+}$ cells (n=6) and $CD8^{+}$ cells (n=6) per field. Data were presented as mean±SD. ns no statistical significance, $^{*}P<0.05$, $^{**}P<0.01$, $^{***}P<0.001$, $^{****}P<0.0001$. shnc short hairpin RNA negative control, sh short hairpin RNA, AOD average optical

density

Figure S1

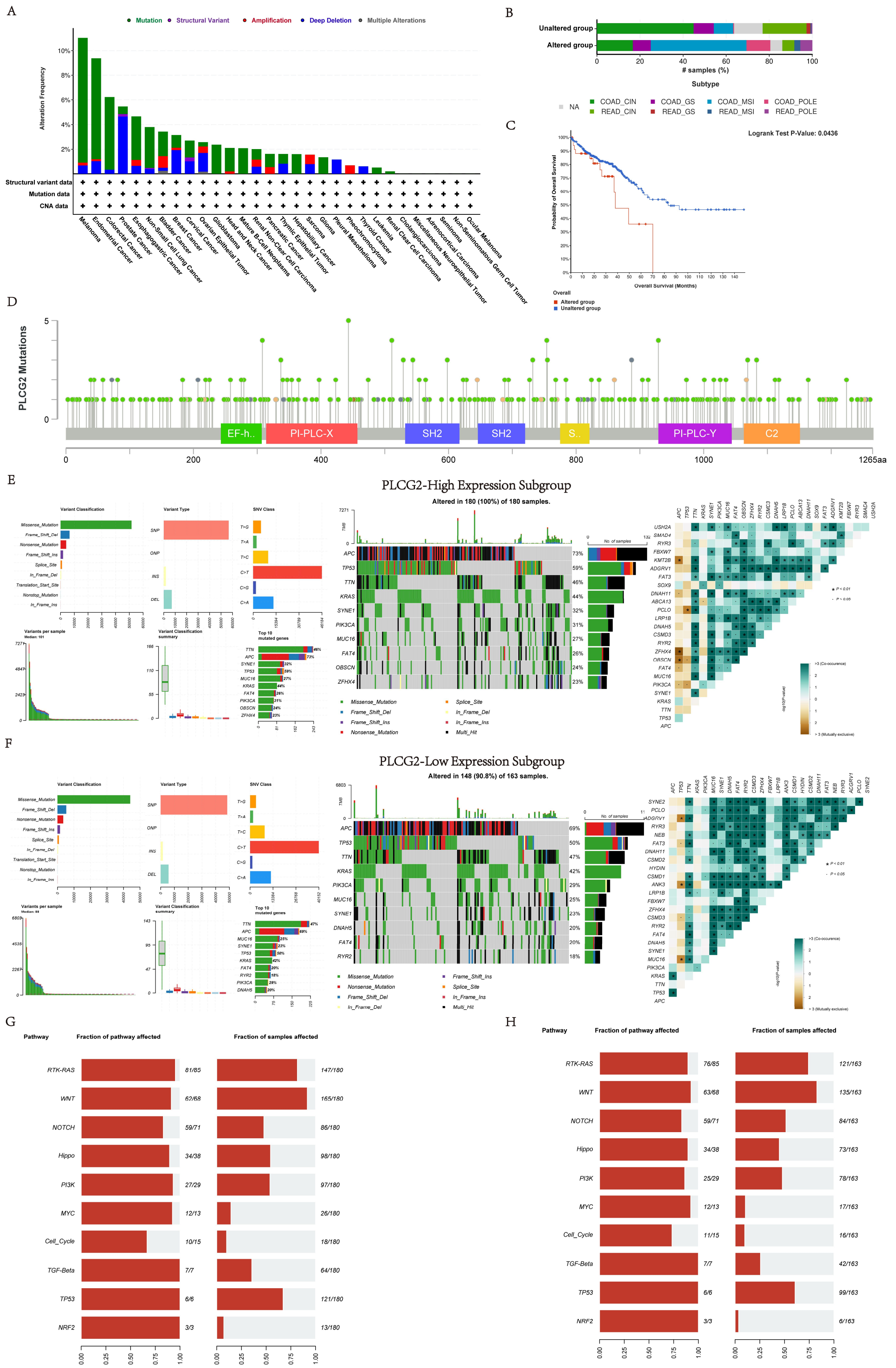

A
B
C
D
E
F
G
H
PLCG2-High Expression Subgroup
Altered in 180 (100%) of 180 samples.
PLCG2-Low Expression Subgroup
Altered in 148 (90.8%) of 163 samples.
Logrank Test P-Value: 0.0436

Figure S2

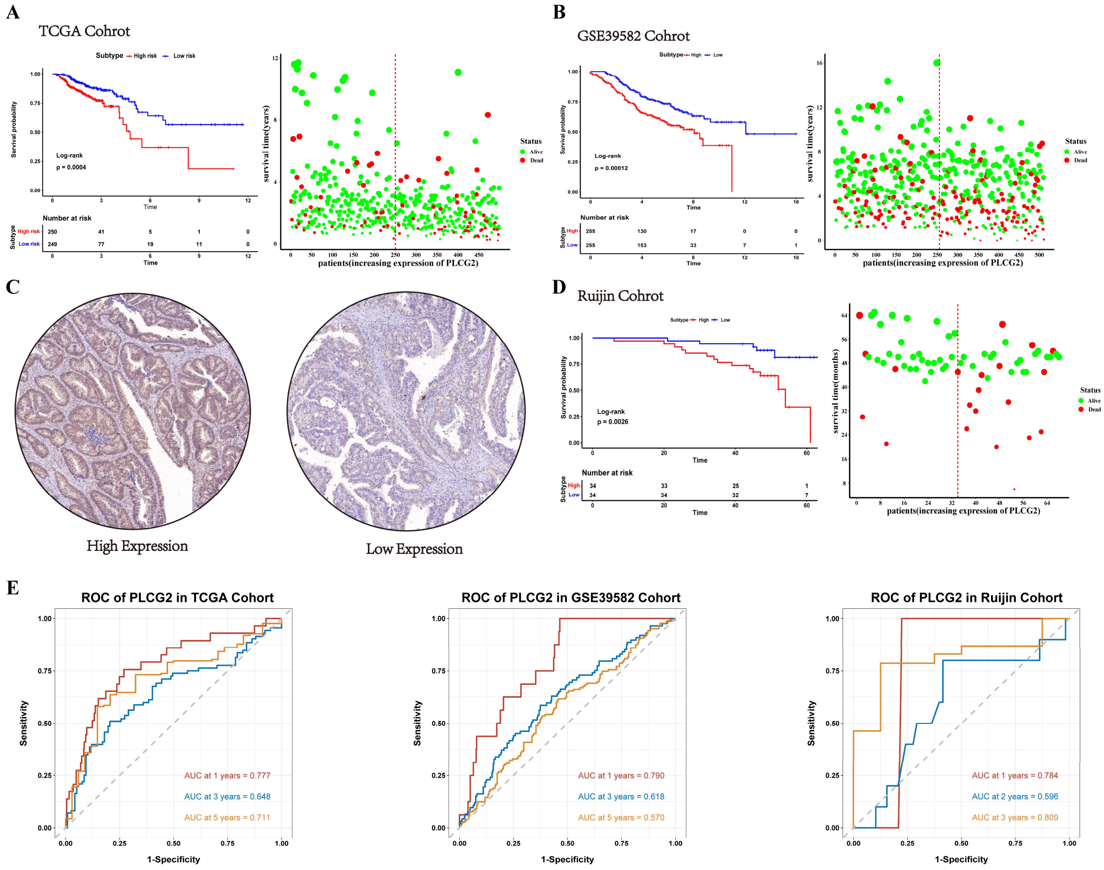

A
TCGA Cohrot
Subtype
High risk
Low risk
Survival probability
Log-rank
p = 0.0004
Time
Number at risk
High risk 250 41 5 1 0
Low risk 249 77 19 11 0
survival time(years)
patients(increasing expression of PLCG2)
Status
Alive
Dead
B
GSE39582 Cohrot
Log-rank
p = 0.00012
High 255 130 17 0 0
Low 255 153 33 7 1
C
High Expression
Low Expression
D
Ruijin Cohrot
Log-rank
p = 0.0026
High 34 33 25 1
Low 34 34 32 7
survival time(months)
E
ROC of PLCG2 in TCGA Cohort
AUC at 1 years = 0.777
AUC at 3 years = 0.648
AUC at 5 years = 0.711
ROC of PLCG2 in GSE39582 Cohort
AUC at 1 years = 0.790
AUC at 3 years = 0.618
AUC at 5 years = 0.570
ROC of PLCG2 in Ruijin Cohort
AUC at 1 years = 0.784
AUC at 2 years = 0.596
AUC at 3 years = 0.809
Sensitivity
1-Specificity

Figure S3

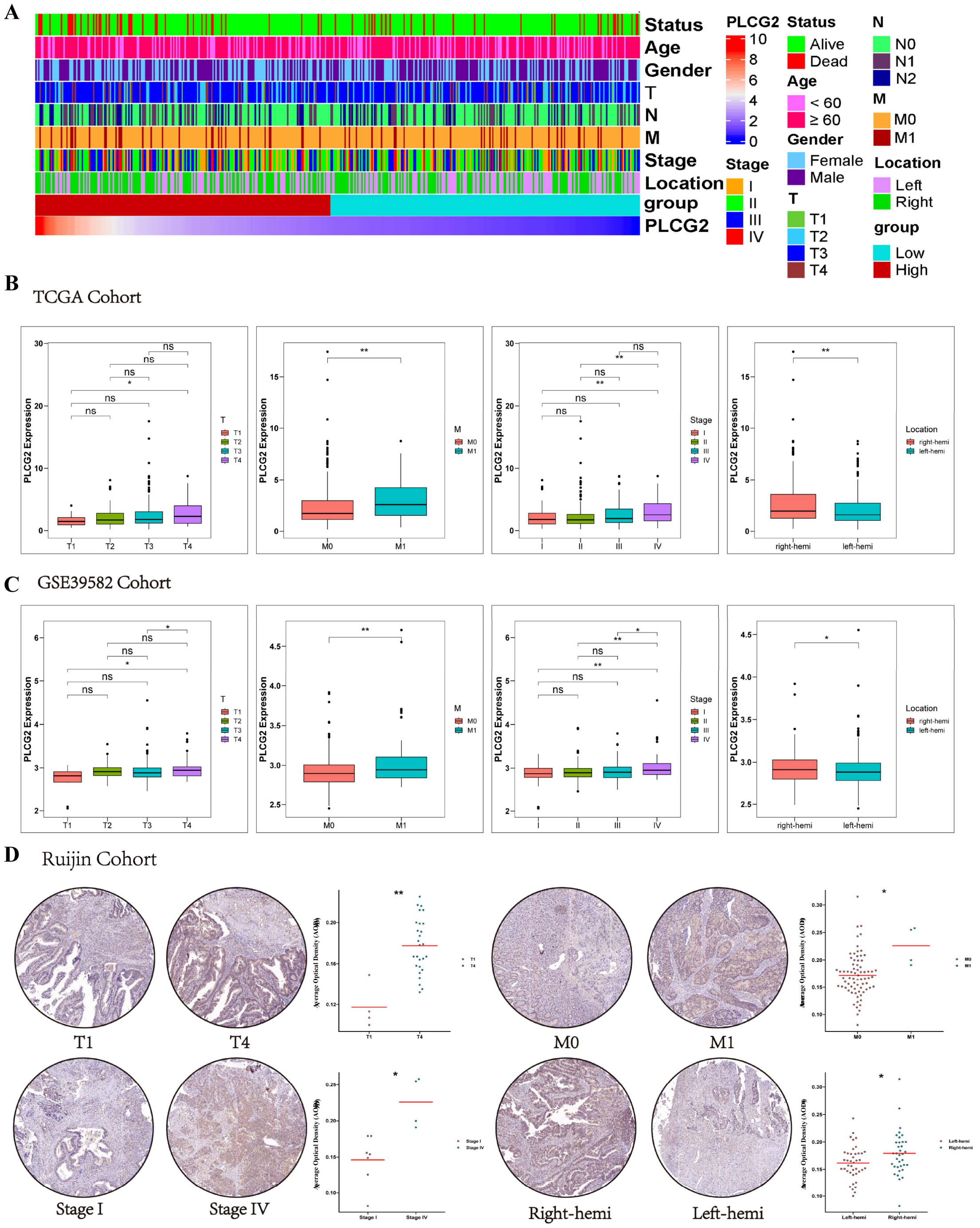

A
Status
Age
Gender
T
N
M
Stage
Location
group
PLCG2
PLCG2
10
8
6
4
2
0
Stage
I
II
III
IV
Status
Alive
Dead
Age
< 60
≥ 60
Gender
Female
Male
T
T1
T2
T3
T4
N
N0
N1
N2
M
M0
M1
Location
Left
Right
group
Low
High
B
TCGA Cohort
PLCG2 Expression
C
GSE39582 Cohort
PLCG2 Expression
D
Ruijin Cohort
T1
T4
M0
M1
Stage I
Stage IV
Right-hemi
Left-hemi

Figure S4

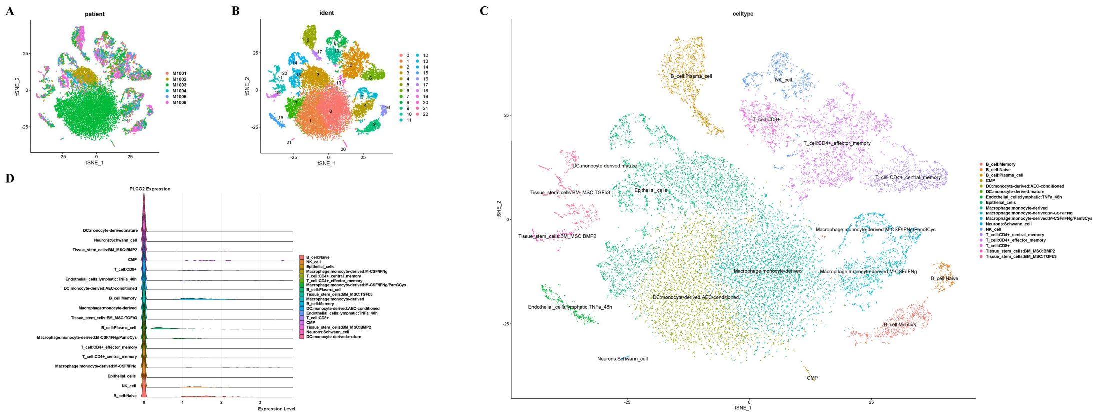
A
patient
M1001
M1002
M1003
M1004
M1005
M1006
tSNE_1
tSNE_2
B
ident
C
celltype
B_cell:Plasma_cell
NK_cell
T_cell:CD8+
T_cell:CD4+_effector_memory
T_cell:CD4+_central_memory
DC:monocyte-derived:mature
Epithelial_cells
Tissue_stem_cells:BM_MSC:TGFb3
Tissue_stem_cells:BM_MSC:BMP2
Macrophage:monocyte-derived
Macrophage:monocyte-derived:M-CSF/IFNg/Pam3Cys
Macrophage:monocyte-derived:M-CSF/IFNg
DC:monocyte-derived:AEC-conditioned
B_cell:Naive
B_cell:Memory
Endothelial_cells:lymphatic:TNFa_48h
Neurons:Schwann_cell
CMP
D
PLCG2 Expression
Expression Level

Figure S5

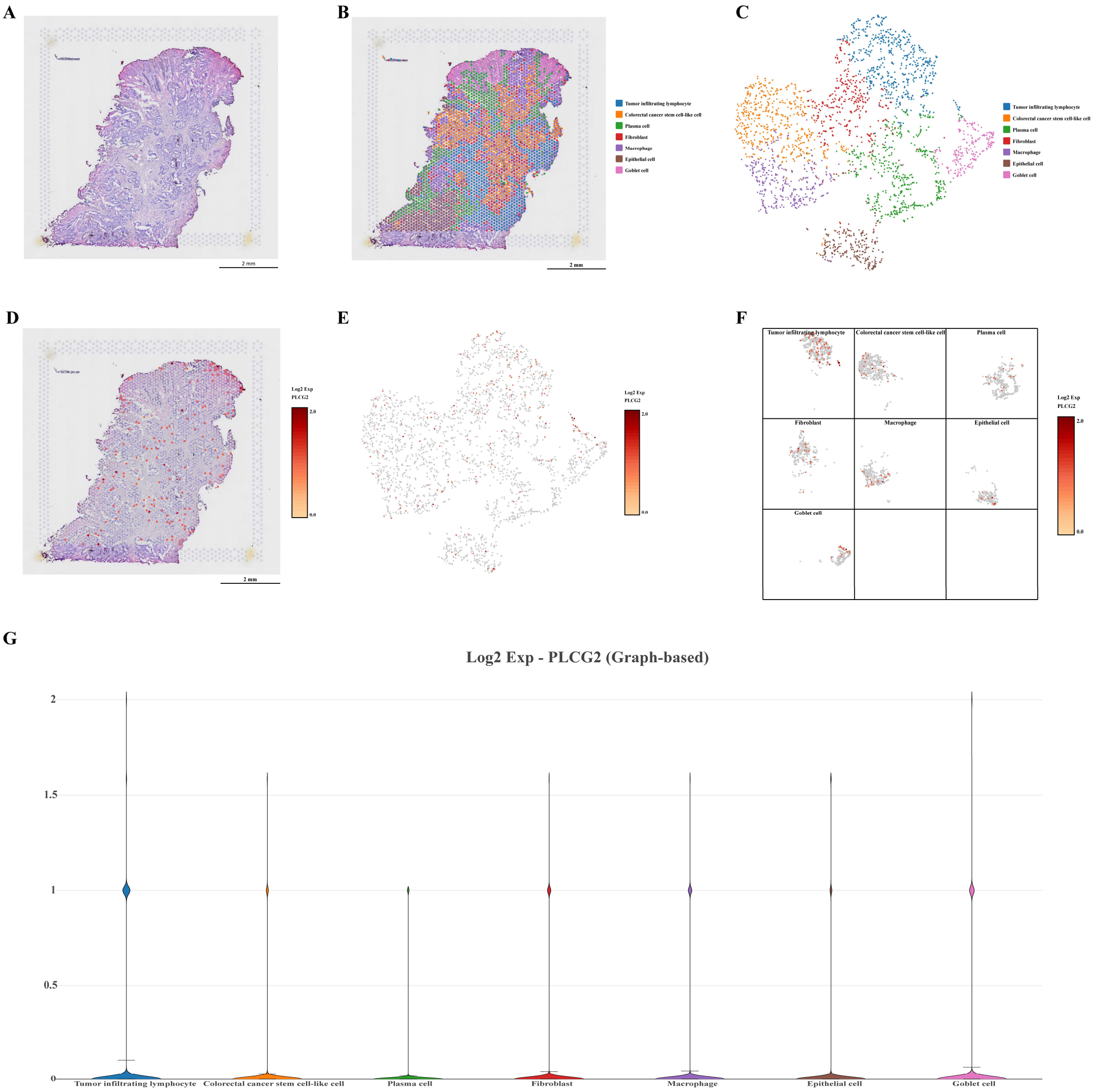
A
B
C
D
E
F
G
2 mm
Tumor infiltrating lymphocyte
Colorectal cancer stem cell-like cell
Plasma cell
Fibroblast
Macrophage
Epithelial cell
Goblet cell
Log2 Exp
PLCG2
2.0
0.0
Log2 Exp - PLCG2 (Graph-based)
2
1.5
1
0.5
0

Figure S6

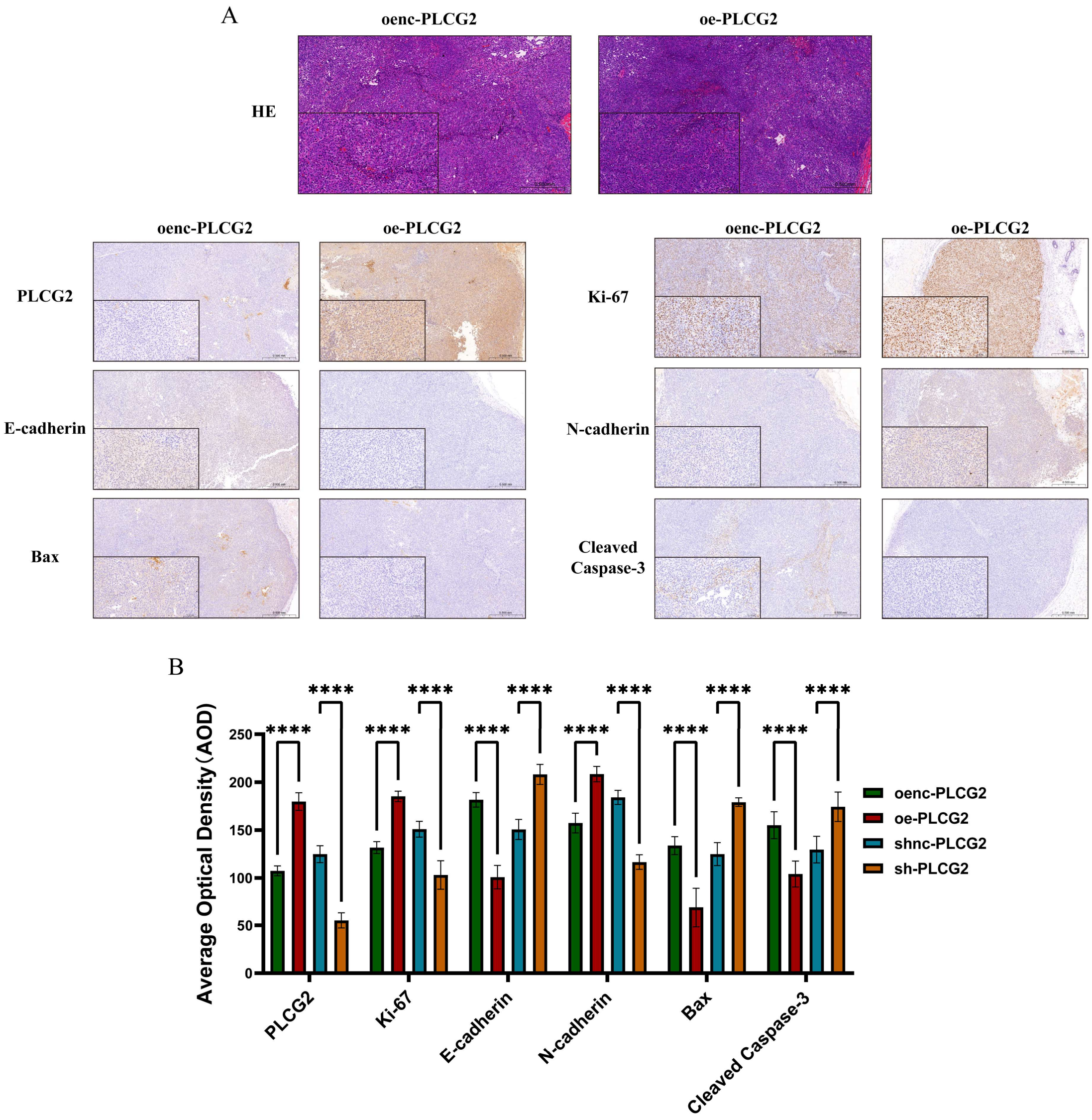
A
oenc-PLCG2
oe-PLCG2
HE
oenc-PLCG2
oe-PLCG2
oenc-PLCG2
oe-PLCG2
PLCG2
Ki-67
E-cadherin
N-cadherin
Bax
Cleaved Caspase-3
B
Average Optical Density (AOD)
250
200
150
100
50
0
****
PLCG2
Ki-67
E-cadherin
N-cadherin
Bax
Cleaved Caspase-3
oenc-PLCG2
oe-PLCG2
shnc-PLCG2
sh-PLCG2

Figure S7

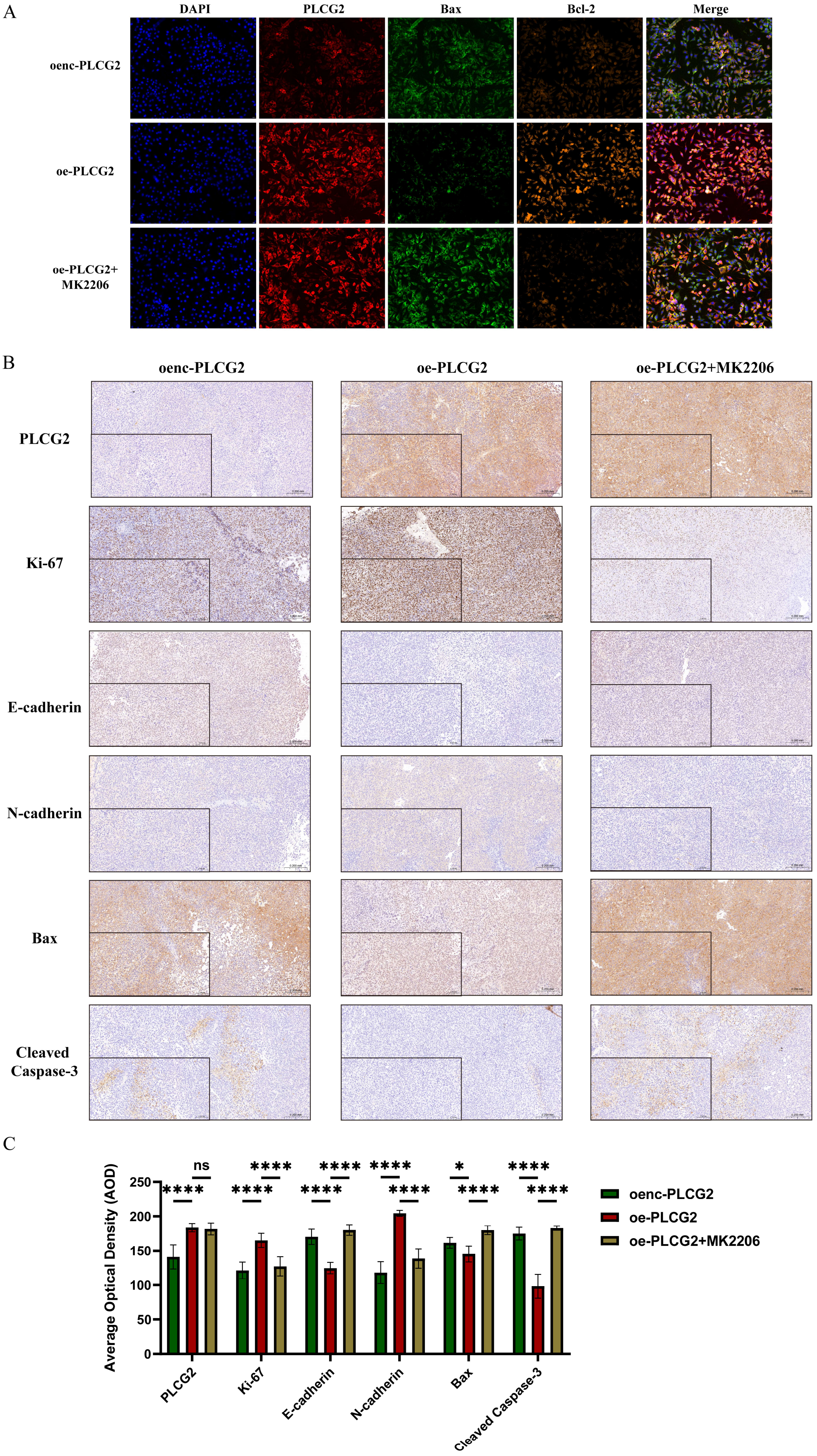
A
DAPI
PLCG2
Bax
Bcl-2
Merge
oenc-PLCG2
oe-PLCG2
oe-PLCG2+
MK2206
B
oenc-PLCG2
oe-PLCG2
oe-PLCG2+MK2206
PLCG2
Ki-67
E-cadherin
N-cadherin
Bax
Cleaved
Caspase-3
C
Average Optical Density (AOD)
250
200
150
100
50
0
ns
****
*
PLCG2
Ki-67
E-cadherin
N-cadherin
Bax
Cleaved Caspase-3
oenc-PLCG2
oe-PLCG2
oe-PLCG2+MK2206

Figure S8

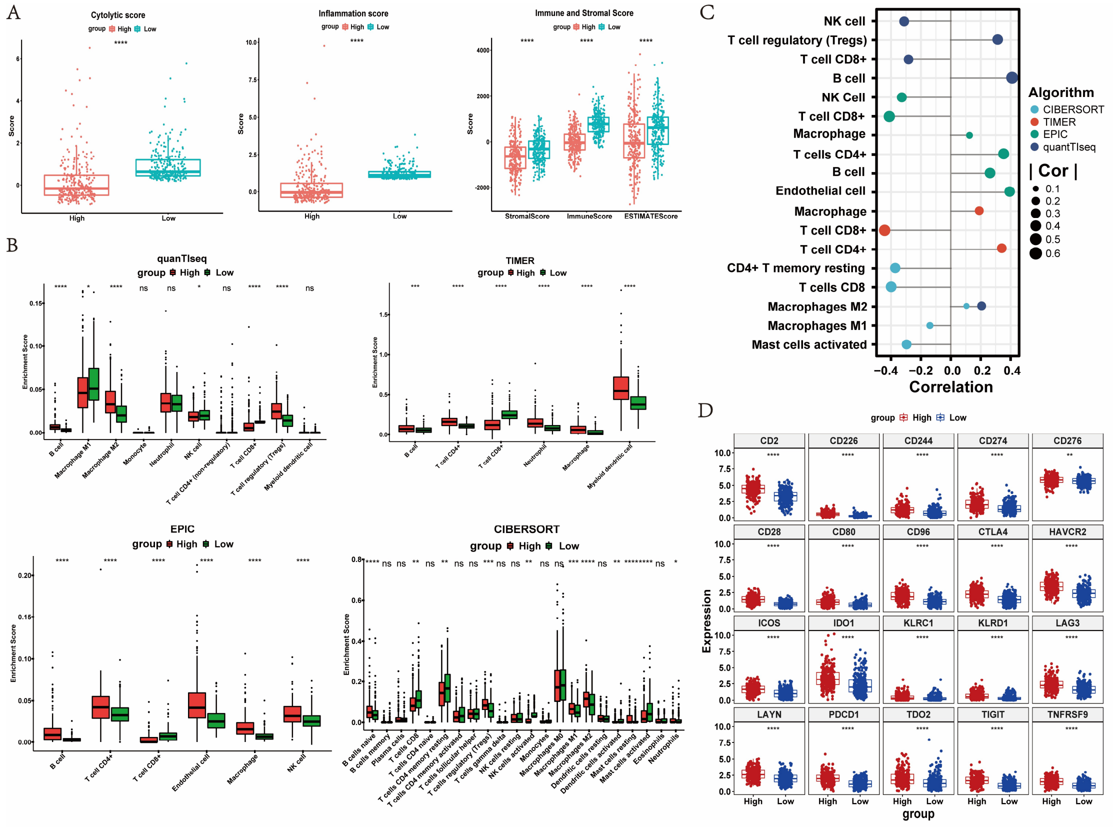

A
Cytolytic score
Inflammation score
Immune and Stromal Score
group High Low
Score
High
Low
StromalScore
ImmuneScore
ESTIMATEScore
B
quanTIseq
TIMER
EPIC
CIBERSORT
Enrichment Score
C
NK cell
T cell regulatory (Tregs)
T cell CD8+
B cell
NK Cell
T cell CD8+
Macrophage
T cells CD4+
B cell
Endothelial cell
Macrophage
T cell CD8+
T cell CD4+
CD4+ T memory resting
T cells CD8
Macrophages M2
Macrophages M1
Mast cells activated
Correlation
Algorithm
CIBERSORT
TIMER
EPIC
quanTIseq
| Cor |
D
CD2
CD226
CD244
CD274
CD276
CD28
CD80
CD96
CTLA4
HAVCR2
ICOS
IDO1
KLRC1
KLRD1
LAG3
LAYN
PDCD1
TDO2
TIGIT
TNFRSF9
Expression
group

Figure S9

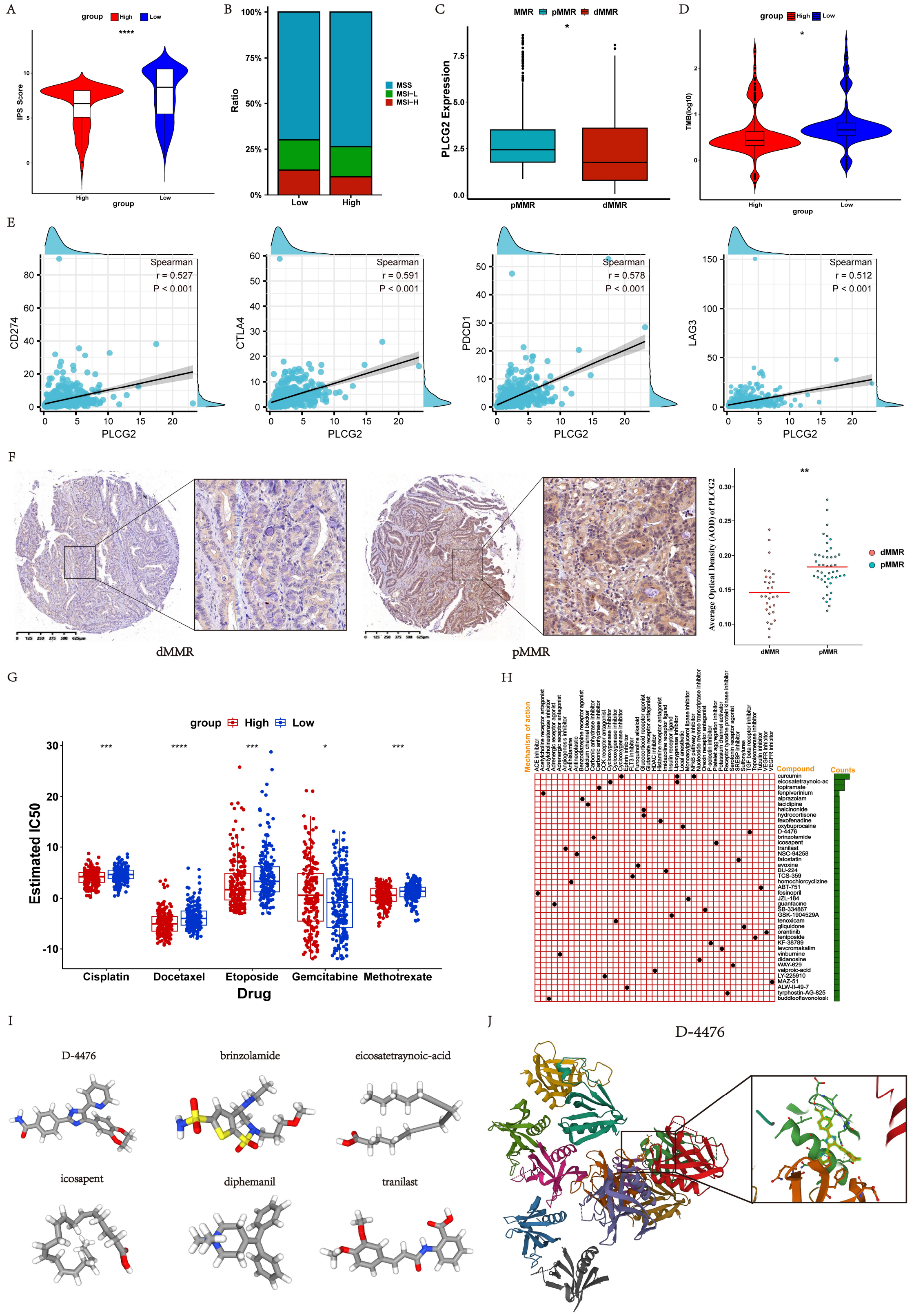

A
B
C
D
E
F
G
H
I
J
group High Low
IPS Score
MSS
MSI-L
MSI-H
Ratio
MMR pMMR dMMR
PLCG2 Expression
TMB(log10)
Spearman
r = 0.527
P < 0.001
r = 0.591
r = 0.578
r = 0.512
CD274
CTLA4
PDCD1
LAG3
PLCG2
dMMR
pMMR
Average Optical Density (AOD) of PLCG2
Estimated IC50
Cisplatin
Docetaxel
Etoposide
Gemcitabine
Methotrexate
Drug
Mechanism of action
Compound
Counts
D-4476
brinzolamide
eicosatetraynoic-acid
icosapent
diphemanil
tranilast

Figure S10

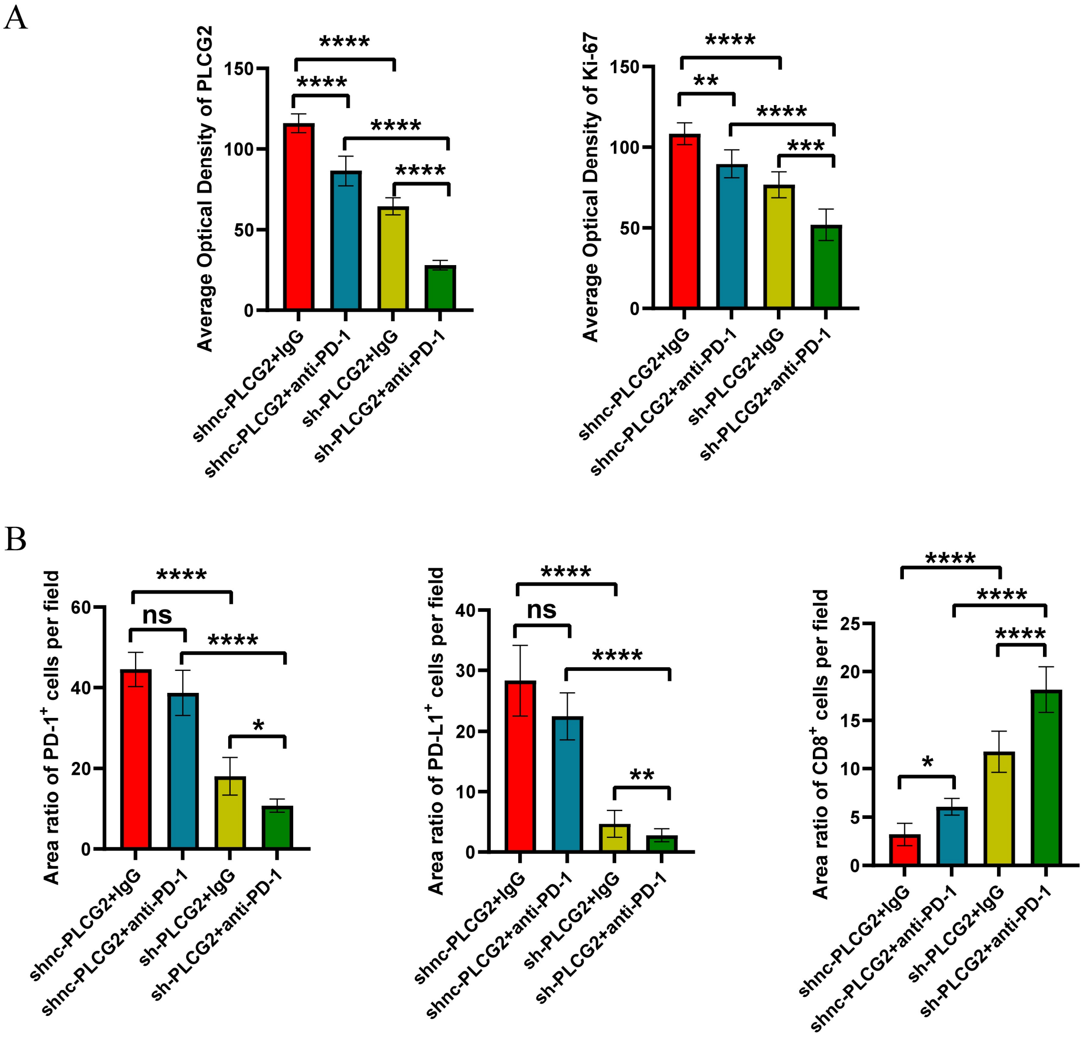
A
Average Optical Density of PLCG2
Average Optical Density of Ki-67
shnc-PLCG2+IgG
shnc-PLCG2+anti-PD-1
sh-PLCG2+IgG
sh-PLCG2+anti-PD-1
B
Area ratio of PD-1⁺ cells per field
Area ratio of PD-L1⁺ cells per field
Area ratio of CD8⁺ cells per field
ns

Table S1 The reagents and resources used in this study.

REAGENT or RESOURCE	SOURCE	IDENTIFIER
**Antibodies for western blotting**		
Human-PLCγ2 Rabbit mAb	Cell Signaling Technology	55512S
Human-E-Cadherin Rabbit mAb	Cell Signaling Technology	3195T
Human-Claudin-1 Rabbit mAb	Cell Signaling Technology	13255T
Human-N-Cadherin Rabbit mAb	Cell Signaling Technology	13116T
Human-Snail Rabbit mAb	Cell Signaling Technology	3879T
Human-Caspase-3 Rabbit mAb	Cell Signaling Technology	14220T
Human-Cleaved Caspase-3 Rabbit mAb	Cell Signaling Technology	9664T
Human-Bcl-2 Rabbit mAb	Cell Signaling Technology	4223T
Human-Bax Rabbit mAb	Cell Signaling Technology	5023T
Human-Akt Rabbit mAb	Cell Signaling Technology	4691T
Human-Phospho-Akt (Ser473) Rabbit mAb	Cell Signaling Technology	4060T
Human-Phospho-Akt (Thr308) Rabbit mAb	Cell Signaling Technology	13038T
Human-mTOR Rabbit mAb	Abcam	ab134903
Human-Phospho-mTOR (Ser2481) Rabbit mAb	Abcam	ab137133
Human-Phospho-mTOR (Ser2448) Rabbit mAb	Abcam	ab109268
Human-β-Tubulin Rabbit mAb	Cell Signaling Technology	2128T
**Antibodies and reagents for IF**		
Human-PLCγ2 Rabbit mAb	Cell Signaling Technology	55512S
DAPI	Servicebio	G1011-10ML
IF555- phalloidin	Servicebio	G1249-100T
Human-N-Cadherin Rabbit mAb	Cell Signaling Technology	13116T
Human-E-Cadherin Rabbit mAb	Cell Signaling Technology	3195T

**Antibodies for IHC and mIHC**		
Human-PLCγ2 Rabbit mAb	Abcam	ab133522
Human- Ki-67 Rabbit mAb	Abcam	ab16667
Human-E-Cadherin Rabbit mAb	Cell Signaling Technology	3195T
Human-N-Cadherin Rabbit mAb	Cell Signaling Technology	13116T
Human-Bax Rabbit mAb	Abcam	ab32503
Human-Cleaved Caspase-3 Rabbit mAb	Cell Signaling Technology	9664T
Human-Bcl-2 Rabbit mAb	Abcam	ab182858
Human-Akt Rabbit mAb	Cell Signaling Technology	4691T
Human-Phospho-Akt (Ser473) Rabbit mAb	Cell Signaling Technology	4060T
Human-Phospho-Akt (Thr308) Rabbit pAb	Proteintech	29163-1-AP
Human-mTOR Rabbit mAb	Cell Signaling Technology	2983T
Human-Phospho-mTOR (Ser2448) Rabbit mAb	Cell Signaling Technology	2976S
Human-Phospho-mTOR (Ser2481) Mouse mAb	Santa Cruz Biotechnology	sc-293132
Human-CD3 Rabbit mAb	Abcam	ab135372
Human-CD8A Rabbit mAb	Abcam	ab237709
Human- FOXP3 Mouse mAb	Abcam	ab20034
Human-CD4 Rabbit mAb	Abcam	ab133616
Human-PD-1 Rabbit mAb	Abcam	ab237728
Human-PD-L1 Rabbit mAb	Abcam	ab237726
Mouse-PLCG2 Rabbit mAb	HUABIO	HA721477
Mouse-Ki-67 Rabbit mAb	Abcam	ab16667
Mouse-PD-1 Rabbit mAb	Abcam	ab214421
Mouse-PD-L1 Rabbit mAb	Cell Signaling Technology	64988T
Mouse-CD8A Rabbit mAb	Cell Signaling Technology	ab237709
**Antibodies and reagents for flow cytometry**		

eBioscience™ Fixable Viability Dye eFluor™ 506	Invitrogen	65-0866-18
FITC anti-mouse CD45	BioLegend	103108
APC-Cy™7 anti-mouse CD3	BD Biosciences	557596
PerCP-Cy™5.5 anti-mouse CD8A	BD Biosciences	551162
Alexa Fluor® 700 anti-mouse Granzyme B	BioLegend	372222
Brilliant Violet 421™ anti-mouse IFN-γ	BioLegend	505830
PE anti-mouse Perforin-1	Invitrogen	12-9392-82
APC anti-mouse CD279 (PD-1)	BioLegend	135209
PE/Cyanine7 anti-mouse TNF-α	BioLegend	506324
PerCP/Cyanine5.5 anti-mouse CD326 (Ep-CAM)	BioLegend	118219
APC anti-mouse CD274 (B7-H1, PD-L1)	BioLegend	124312
